# Identification of Clinical Response Predictors of Tocilizumab Treatment in Patients with Severe COVID-19 Based on Single-Center Experience

**DOI:** 10.3390/jcm12062429

**Published:** 2023-03-22

**Authors:** Wiktor Schmidt, Katarzyna Pawlak-Buś, Barbara Jóźwiak, Piotr Leszczyński

**Affiliations:** 1 Department of Rheumatology, Systemic Connective Tissue Diseases and Immunotherapy of Rheumatic Diseases, J. Strus Municipal Hospital, 61-285 Poznan, Poland; 2 Department of Internal Medicine, Poznan University of Medical Sciences, 61-701 Poznan, Poland; 3 Doctoral School, Poznan University of Medical Sciences, 60-812 Poznan, Poland

**Keywords:** COVID-19, SARS-CoV-2, COVID-19 drug treatment, interleukin-6, immunomodulation

## Abstract

Hyperinflammation in COVID-19 plays a crucial role in pathogenesis and severity; thus, many immunomodulatory agents are applied in its treatment. We aimed to identify good clinical response predictors of tocilizumab (TCZ) treatment in severe COVID-19, among clinical, laboratory, and radiological variables. We conducted a prospective, observational study with 120 patients with severe COVID-19 not improving despite dexamethasone (DEX) treatment. We used parametric and non-parametric statistics, univariate logistic regression, receiver operating characteristic (ROC) curves, and nonlinear factors tertile analysis. In total, 86 (71.7%) patients achieved the primary outcome of a good clinical response to TCZ. We identified forty-nine predictive factors with potential utility in patient selection and treatment monitoring. The strongest included time from symptom onset between 9 and 12 days, less than 70% of estimated radiological lung involvement, and lower activity of lactate dehydrogenase. Additional predictors were associated with respiratory function, vitamin D concentration, comorbidities, and inflammatory/organ damage biomarkers. Adverse events analysis proved the safety of such a regimen. Our study confirmed that using TCZ early in the hyperinflammatory phase, before severe respiratory failure development, is most beneficial. Considering the described predictive factors, employing simple and widely available laboratory, radiological, and clinical tools can optimize patient selection for immunomodulatory treatment with TCZ.

## 1. Introduction

Hyperinflammation with autoimmune and autoinflammatory features was noticed early in the severe acute respiratory syndrome coronavirus 2 (SARS-CoV-2) pandemic, especially in severe and critical patients [1]. The resemblance of critical coronavirus disease 2019 (COVID-19) to cytokine storm syndromes, a well-described group of numerous clinical identities, was stated and confirmed [2,3]. Infections are the most common cause of cytokine storm syndromes, with bacterial infections inducing a sepsis-related cytokine storm and viral infections being the leading source of secondary hemophagocytic lymphohistiocytosis (sHLH) [4]. Serum cytokine profiles assessed by Huang et al. in severe COVID-19 were similar to those found in sHLH [5]. Early reports from the COVID-19 pandemic on mortality predictors found elevated interleukin-6 (IL-6), C-reactive protein (CRP), and ferritin serum concentrations that were significant [6]. They were associated with disease severity and prognosis, which was replicated in many further papers [7]. Autopsy studies confirmed that an excessive immune response leads to irreversible endothelial, lung, and multi-organ damage, independently of viral replication and a direct cytopathic effect [8,9,10,11]. Hemophagocytosis was found to be present in the bone marrow and reticuloendothelial organs of patients with COVID-19 and elevated inflammatory markers (CRP, ferritin, IL-6, IL-8, and TNF-α), similarly to sHLH. It is induced by activated alveolar macrophages. Significant autopsy-proven organ damage in these patients involved the lungs, spleen, lymph nodes, central nervous system, heart, kidneys, liver, and pancreas [12]. However, some differences between severe COVID-19 and other cytokine storm syndromes were noted, such as lower levels of circulating cytokines, rarer multisystem manifestations, and worse outcomes of steroid monotherapy than in severe acute respiratory syndrome coronavirus 1 (SARS-CoV-1) and Middle East respiratory syndrome-related coronavirus (MERS-CoV) infections [13,14]. Thus, hyperinflammation in COVID-19 seems to be a distinct clinical entity and cannot be classified with criteria developed for other cytokine storm syndromes. Considering endothelial damage with immunothromboembolism, it was either as COVID-19-associated hyperinflammatory syndrome or pulmonary intravascular coagulation [15,16,17]. Based on the abovementioned rationale, immunosuppressive and immunomodulatory therapies were tested in numerous COVID-19 clinical trials, not without concerns about secondary infections and compromising the proper antiviral response [18]. Tocilizumab (TCZ) is a humanized monoclonal antibody against the interleukin-6 receptor (IL-6R), applied in severe COVID-19 with hyperinflammation since early 2020 [19,20,21]. It was proven to reduce in-hospital mortality, the need for mechanical ventilation, or intensive care unit (ICU) transfer [22,23,24,25,26]. It was approved for use in COVID-19 by the European Medicines Agency (EMA) in December 2021 and one year later by the Food and Drug Administration (FDA).

A clear distinction between a proper inflammatory response from excessive cytokine production using methods available in standard clinical practice remains challenging. In randomized clinical trials (RCTs), most inclusion criteria contained elevated inflammatory markers and hypoxemia—alone or combined [27,28,29]. Results from RCTs are noncoherent, emphasizing the need to qualify patients with COVID-19 for TCZ therapy in a more tailored way [29,30,31,32]. Early administration in the course of the progressive hypoxemic disease seems to be most beneficial [27,28]. Other clinical and laboratory parameters in patient selection are understudied. Moreover, most RCTs focused in their primary points on mortality or ICU transfer, without defining the clinical response. Thus, our study aimed at the analysis of clinical and laboratory response predictors to TCZ treatment added to dexamethasone (DEX) in severe deteriorating COVID-19. We hypothesized that TCZ initiation early in the hyperinflammatory phase of severe COVID-19 would be of the greatest benefit.

## 2. Materials and Methods

### 2.1. Study Setting, Design, and Population

We conducted a single-center, prospective, longitudinal cohort study in 120 adult patients hospitalized due to severe COVID-19 refractory to initial intravenous DEX therapy and qualified to be treated with TCZ. Our ward functioned as a COVID-19 facility from 16 March 2020 to 28 February 2022. We treated predominantly patients with severe COVID-19 pneumonia in need of oxygen supplementation therapy. The first TCZ treatment in severe COVID-19 as a salvage therapy in our ward was instituted on 19 April 2020. The dosing scheme included two doses of 8 mg/kg TCZ intravenously 24 h apart. Such a dosing regimen differed from the standard rheumatoid arthritis (RA) intravenous dosing regimen and was adopted from early reports of its use in COVID-19 and pathogenetic similarities to cytokine storm syndrome observed after chimeric antigen receptor T (CAR-T) cell immunotherapy of hematologic diseases [33] or systemic autoinflammatory diseases [34,35]. Local guidelines for SARS-CoV-2 infection treatment have recommended the use of TCZ in severe COVID-19 (up to two intravenous doses of 8 mg/kg 8–24 h apart) since 31 March 2020 and they recommend it up until the latest version [36,37,38].

A study was conducted in our ward (multispecialty hospital, secondary care setting) between 4 February 2021 and 31 December 2021. A total of 120 consecutive patients with severe COVID-19 refractory to intravenous DEX treatment (initiated in Emergency Ward) were qualified by rheumatologists/internists to be treated with tocilizumab. Severe COVID-19 was defined as meeting all three criteria [39,40]:

(1) Positive real-time reverse transcription–polymerase chain reaction test (RT-PCR) for SARS-CoV-2 from nasopharyngeal swab on admission;
(2) Interstitial pneumonia defined as the presence of ground-glass opacities present in lung chest computed tomography (CT) scans;
(3) Hypoxemia defined as peripheral blood saturation measured with pulse oximeter (SpO_2_) < 94%.

Patients refractory to DEX treatment (4–8 mg intravenously every 12 h) were defined as those whose respiratory function did not improve after two doses. They must have met both of the two criteria:

(1) No improvement in SpO_2_/fraction of inspired oxygen (FiO_2_) and
(2) No decrease in oxygen supplementation demand (measured as oxygen flow, QO_2_, L/min).

Rheumatologists with at least 10 years of experience with TCZ use in autoimmune disorders assigned patients to TCZ treatment after the initial abovementioned criteria fulfillment. Analysis of additional clinical, radiological, and laboratory data and the exclusion of active, severe infections other than COVID-19 were performed. Each patient received two doses of 8 mg/kg of intravenous TCZ 24 h apart, due to evidence mentioned previously and our previous experience in severe COVID-19 treatment. 

The study size was arrived at by including all eligible patients hospitalized within the study time range. Patients were followed through hospitalization until discharge or death. Apart from TCZ, severe COVID-19 cases were treated following current local guidelines regarding SARS-CoV-2 infection treatment and their updates published as supplements [36]. Oxygen flow was titrated to achieve SpO_2_ of 95–98%. If a patient deteriorated despite low-flow oxygen supplementation, a high-flow nasal cannula (HFNC) was used. If the clinical status worsened despite this, they consulted with anesthesiologists and were qualified for ICU treatment. Thromboembolic prophylaxis was administered according to local guidelines and their updates [41]. 

The study was developed and described in accordance with the Strengthening the Reporting of Observational Studies in Epidemiology (STROBE) statement: guidelines for reporting observational studies [42,43]. The Ethical Committee of Poznan University of Medical Sciences approved the study on 4 February 2021 (Consent No. 108/21).

### 2.2. Clinical, Radiological, and Laboratory Assessment

We collected baseline clinical and demographical data during admission: age, sex, vaccination status (patients vaccinated were defined as those who received ≥2 doses of mRNA-1273 or BNT162b2 vaccine or ≥1 dose of Ad26.COV2.S), infection type (primary or reinfection), clinical symptoms, their onset and duration, comorbidities and Charlson Comorbidity Index (CCI) [44], current treatment, body weight, height, and body mass index (BMI). Other clinical parameters were gathered prospectively throughout hospitalization and they included temperature, systolic blood pressure, diastolic blood pressure, heart rate, respiratory rate, SpO_2_ measured with finger pulse oximeter, FiO_2_ estimated with Wettstein method [45], QO_2_, diuresis, mental status assessment (Alert, Response to Voice, Response to Pain, Unresponsive—AVPU—and Alert, Confused, Drowsy, Unresponsive—ACDU) [46], Modified Early Warning Score (MEWS) [47], Quick Sepsis-Related Organ Failure Assessment (qSOFA) score [48,49], World Health Organization (WHO) clinical progression scale known also as the WHO Ordinal Scale [50], ROX index (division of SpO_2_/FiO_2_ index in % by respiratory rate in breaths/min) [51], modified ROX index (HR-ROX index; ratio of ROX index over HR (beats/min), multiplied by a factor of 100) [52], and SpO_2_/FiO_2_ index [53]. They were assessed five times during the study:

- Baseline data—from Emergency Department (ED) or from ward admission;
- On TCZ initiation;
- On 2nd day after TCZ first dose;
- On 5th day after TCZ first dose;
- Last reported data—before discharge or death.

Each patient had initial chest CT obtained during hospital admission (in ED or during ED–ward transfer) with a Siemens Somatom Sensation 64-slice computed tomography machine. A high-resolution chest CT protocol without intravenous contrast was applied (slice thickness 1.0 mm, 120 kV, 150 mA, 0.5 s rotation time, pitch 0.9, kernel B80f, detector collimation 0.6 mm, matrix 512 × 512 mm). The percentage of opacity (total percent of affected lung parenchyma) was analyzed. Pulmonary and mediastinal windows were acquired. Radiological aberrances were reported per the Fleischner Society glossary [54]. Lung parenchyma involvement and COVID-RRS were calculated by a radiologist with >15 years of experience in chest CT [55], using Frontier Lung Analysis software (Siemens, Erlangen, Germany). Reconstruction with the Br59 kernel was used.

All laboratory results were obtained using laboratory analyzers—Roche Cobas c501, Siemens ADVIA Centaur CP, Abbott ARCHITECT i1000SR, Sysmex XN-100,0, and Werfen ACL Top 700—in our in-hospital laboratory. Baseline laboratory parameters were obtained on admission (in ED or during 1st day of ward stay) and they consisted of tests performed only on admission:

- Arterial blood gas with arterial lactate concentration;
- Fasting glucose concentration in venous blood;
- Thyrotropic hormone (TSH) serum activity;
- Calcifediol (25(OH)D_3_) serum concentration.

Then, basic laboratory tests were performed at least 5 times during hospitalization (at the same abovementioned time points as basic clinical parameters):

- Complete blood count (CBC) with automated differential and complex indices derived from it: neutrocyte-to-lymphocyte ratio (NLR) and neutrocyte-to-monocyte ratio (NMR), and immature neutrocyte count (INC) analysis;
- Inflammatory parameters: C-reactive protein (CRP), interleukin-6 (IL-6) and procalcitionin (PCT) serum concentration;
- Electrolytes and renal parameters: sodium and potassium serum concentration, blood urea nitrogen (BUN), serum creatinine (SCr);
- Serum liver enzymes: aspartate aminotransferase (AST), alanine aminotransferase (ALT), total bilirubin;
- Organ damage indices: D-dimer serum concentration, lactate dehydrogenase (LDH) serum activity.

Laboratory parameters tested four times during the study (baseline, on TCZ initiation, on the 5th day after TCZ initiation, and before discharge/death) included

- Fibrinogen serum concentration;
- Organ damage indices: high sensitive troponin I (hs-TnI);
- Coagulation parameters: international normalized ratio (INR), prothrombin time (PT), activated partial thromboplastin time (aPTT).

Tests performed three times during the study (on TCZ initiation, on the 5th day after TCZ initiation, and before discharge/death) were as listed:

- Ferritin serum concentration;
- Serum liver enzyme activity: alkaline phosphatase (ALP), gamma-glutamyl transferase (GGT);
- High sensitive troponin I (hs-TnI).

Additional tests were performed two times during the study (on TCZ initiation and on the 5th day after it):

- Electrolytes and renal parameters: urinalysis, chloride;
- Organ damage indices: creatine kinase (CK) serum activity, B-natriuretic peptide (BNP) serum concentration;
- Total protein (TP) and albumin serum concentration.

### 2.3. Outcomes

The primary outcome was a good clinical response to tocilizumab therapy, defined as meeting 2 of 2:

(1) Respiratory improvement on 5th day after first TCZ infusion compared to the day of treatment initiation, defined by elevation of SpO_2_/FiO_2_ of at least 20;
(2) No further respiratory deterioration after 5th day from TCZ initiation until hospital discharge, and no progression to ICU or death during hospitalization.

Additional outcomes of interest were analyzed to assess the safety and effectiveness of TCZ treatment. They were as follows:

- Death;
- Intensive care unit (ICU) transfer due to acute respiratory distress syndrome (ARDS) progression [56];
- Respiratory deterioration (based on SpO_2_/FiO_2_ index);
- Venous thromboembolic disease: pulmonary embolism (PE) [57], deep vein thrombosis (DVT) [58];
- Hematological manifestations: COVID-19-associated coagulopathy (CAC) [59], disseminated intravascular coagulation (DIC) [60,61], severe lymphopenia, hemolytic anemia, neutropenia, agranulocytosis, thrombocytopenia and severe thrombocytopenia, minor and major bleeding episodes [62];
- Cardiovascular manifestations: major adverse cardiovascular events (MACE) [63] including acute myocardial injury (AMI) [64], acute coronary syndromes (ACS), myocarditis, new onset of atrial fibrillation (AF), chronic heart failure decompensation (HF) or HF de novo, stroke and embolism; hypotension and hemodynamic instability;
- Hepatic outcomes: liver injury and its type [65], drug-induced liver injury (DILI) [66], severe liver dysfunction (SLD) [67];
- Secondary infections, including sepsis and septic shock [68];
- Kidney outcomes: acute kidney injury (AKI) [69], AKI demanding renal replacement therapy (RRT), acute tubulointerstitial nephritis, proteinuria, sterile pyuria;
- Neuropsychiatric manifestations: acute confusional state, COVID-19 encephalopathy [70];
- Complex inflammatory outcomes and indices: COVID-19 hyperinflammation syndrome (COV-HI) [16], COVID-19-associated hyperinflammation syndrome (cHIS) and cHIS score [71], and COVID-19 cytokine storm (CCS) [72].

Definitions of additional outcomes are available in Supplementary Table S1.

### 2.4. Statistical Analysis

Statistical analysis was conducted with PQStat v. 1.8.2 (PQStat Software, Poznan, Poland) and figures were prepared with GraphPad Prism v. 9.5.0 (GraphPad Software, Boston, MA, USA). Categorical data were compared with the χ^2^ test when Cochrane’s rule was applicable, and otherwise with Fisher’s exact test. Continuous data with normal distributions were analyzed with the *t*-Student test (if equality of variances was stated). The Mann–Whitney U test was applied in comparing ordinal and continuous data without normal distribution. Receiver operating characteristic (ROC) curves of potential predictors were developed and cut-off points of were achieved with the Youden index. Afterwards, a univariate analysis using logistic regression was conducted to calculate odds ratios (OR) of predictors. To avoid omitting potential predictors without a linear distribution, additional tertile analysis of variables was performed with OR graph analysis and derivation of ranges of nonlinear predictors. Statistical significance was defined as a *p*-value of 0.05 or less.

## 3. Results

### 3.1. Demographic and Clinical Characteristics

A total of 120 (57.6%) out of 208 patients with severe COVID-19 hospitalized in our ward during the study were treated with tocilizumab. We identified 86 (71.7%) good clinical responders, and 34 (28.3%) non-responders, with 58.8% (*n* = 20) in-hospital mortality in the latter group. Baseline demographic and clinical characteristics including comorbidities, symptoms and time of their duration, radiological assessment, and instituted treatment are presented in Table 1. Full characterization is provided in Supplementary Table S2.

**Figure 1:**
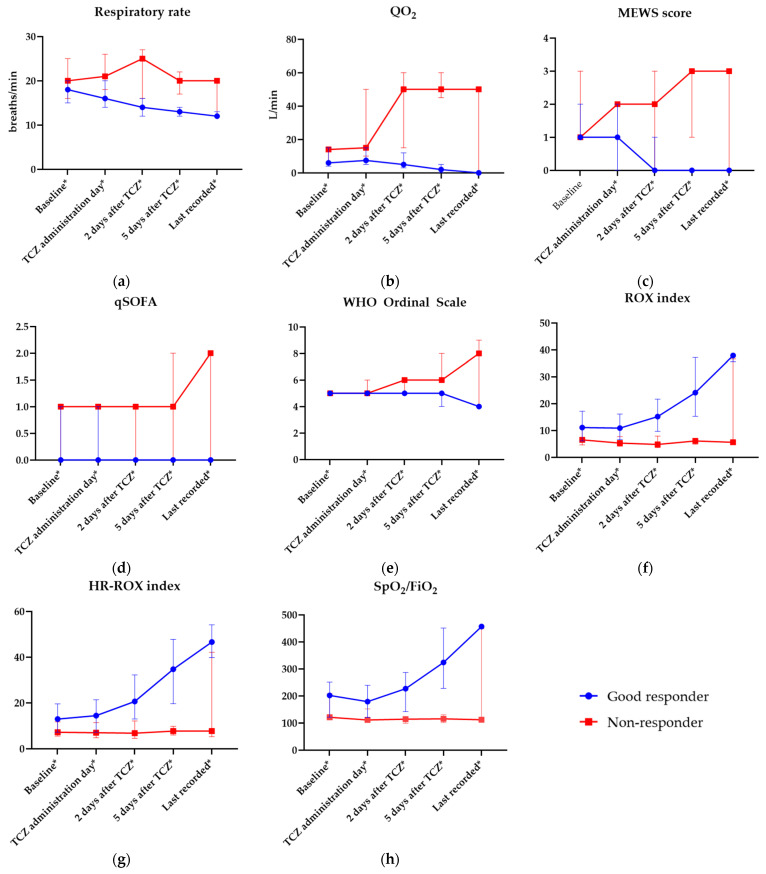
Clinical parameters and complex indices measured during hospitalization in clinical (good) responders and non-responders. Statistically significant differences are bolded and marked with *. (**a**) Respiratory rate (breaths/min); (**b**) oxygen flow (QO_2_, L/min); (**c**) Modified Early Warning Score (MEWS); (**d**) Quick Sepsis-Related Organ Failure Assessment (qSOFA) score; (**e**) World Health Organization (WHO) Ordinal Scale; (**f**) ROX index; (**g**) modified ROX index (HR-ROX); (**h**) ratio of SpO_2_—peripheral oxygen saturation and FiO_2_—fraction of inspired oxygen. Points represent median values of variables and whiskers reflect interquartile ranges (IQR).

Dynamics of change in clinical parameters and complex indices measured throughout the hospitalization that differed significantly between study groups are presented in Figure 1a–h. Full clinical data including all the measured parameters and their values in responder and non-responder groups are available in Supplementary Table S3.

We identified nineteen clinical and radiological predictors of the clinical response before the tocilizumab treatment of severe COVID-19 (obtained via univariate logistic regression analysis). They are presented in Table 2.

### 3.2. Laboratory Analysis

**Figure 2:**
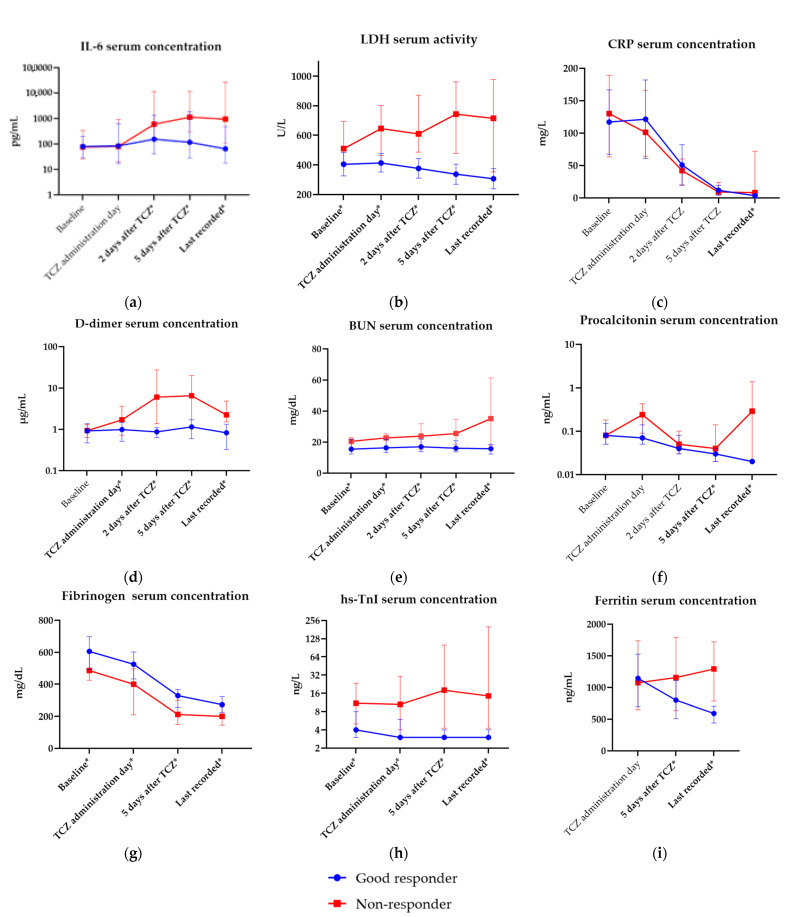
Selected aberrations in laboratory parameters measured throughout hospitalization. Statistically significant differences are bolded and marked with *. (**a**) Interleukin-6 (IL-6) serum concentration (pg/mL); (**b**) lactate dehydrogenase (LDH) serum activity (IU/L); (**c**) C-reactive protein (CRP) serum concentration (mg/L); (**d**) D-dimer serum concentration (µg/mL); (**e**) blood urea nitrogen (BUN) serum concentration (mg/dL); (**f**) procalcitonin serum concentration (ng/mL); (**g**) fibrinogen serum concentration; (**h**) high sensitive troponin I serum concentration (ng/L); (**i**) ferritin serum concentration (ng/mL). Points represent median values of variables and whiskers reflect interquartile ranges (IQR).

**Figure 3:**
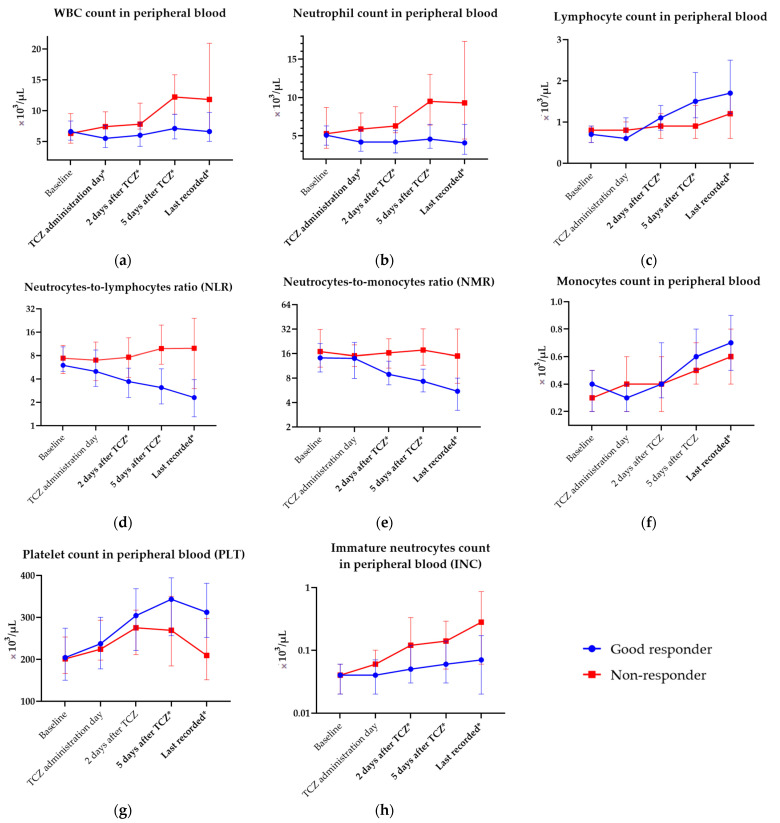
Selected aberrations in complete blood count (CBC) and complex indices measured throughout hospitalization in clinical (good) responders and non-responders. Statistically significant differences are bolded and marked with *. (**a**) White blood cell count (WBC, ×10^3^/µL); (**b**) neutrophil count in peripheral blood (×10^3^/µL); (**c**) lymphocyte count in peripheral blood (×10^3^/µL); (**d**) monocyte count in peripheral blood (×10^3^/µL); (**e**) neutrocyte-to-lymphocyte ratio (NLR); (**f**) neutrocyte-to-monocyte ratio (NMR); (**g**) platelet count in peripheral blood (×10^3^/µL); (**h**) immature neutrocyte count in peripheral blood (INC, ×10^3^/µL). Points represent median values of variables and whiskers reflect interquartile ranges (IQR).

Full laboratory data, including measurements and their values in responder and non-responder groups during hospitalization, are available in Supplementary Table S4. Dynamics of change in selected laboratory parameters that differed significantly between groups are presented in Figure 2a–i. Aberrations in complete blood count (CBC) and complex indices derived from CBC parameters throughout hospitalization are shown in Figure 3a–h.

In the univariate logistic regression and tertile analysis of nonlinear variables, we identified twelve predictors of the clinical response to TCZ in severe COVID-19 among laboratory parameters. They are listed in Table 3.

### 3.3. Early Prediction of Good Outcome after Tocilizumab Initiation and Mortality Prediction

Analysis of the parameters measured on the second day after TCZ initiation provided 18 additional predictors, presented in Table 4. Mortality predictors and their comparisons to good response prediction in our study are listed in Table 5.

### 3.4. Additional Outcomes and Safety

All outcomes of interest were analyzed on their prevalence upon TCZ administration and their later occurrence during the hospitalization. Twenty (58.8%) patients in the non-responder group needed ICU transfer and 19 (55.8%) of them died there. The main causes of death in the ICU were severe ARDS refractory to invasive ventilation and consecutive multiorgan failure (early deaths, within 1–6 days from transfer; *n* = 9) and secondary infections, predominantly A. baumanii ventilator-associated pneumonia (VAP) progressing to sepsis and septic shock (late deaths, >6 days from ICU admission; *n* = 6). One patient died due to sudden cardiac arrest associated with pulmonary embolism on the ward. There were no deaths or ICU transfers in the clinical responder group per definition. Table 6 presents the outcome summary. All outcomes of the study are available in Supplementary Table S5.

## 4. Discussion

We identified, analyzed, and described thirty-one clinical, radiological, and laboratory good response predictors for TCZ treatment assessed before its administration as an addition to DEX in severe deteriorating COVID-19. We also found eighteen predictive factors of the same outcome assessed early (on second day) after TCZ initiation. Our definition of a good clinical response was derived predominantly from our previous experience with severe COVID-19 and its treatment with immunomodulatory agents (TCZ, DEX, baricitinib). Overall, 71.7% of patients achieved a good clinical response with respiratory function improvement. The mortality rate in the studied group was 16.7% and secondary infections occurred in 13.3% after TCZ initiation, which is comparable to data reported in earlier trials [73]. Respiratory deterioration between TCZ administration and the fourth day after it was noted significantly more often in the non-responder group than in clinical responders (76.5% vs. 15.1%). Our results can affect the optimization of patient selection and treatment monitoring of TCZ in severe COVID-19 patients, as they improved our team’s qualification and the wider use of immunomodulatory agents during the delta variant peak.

Tocilizumab reduces severe COVID-19-associated mortality and the need for mechanical ventilation in a time-dependent way. In a systematic review and meta-analysis by Petrelli et al., the authors found that the reduction in mortality when controlled for other variables was 57% and the need for mechanical ventilation was lowered by 74% [26]. We have confirmed earlier reports of the beneficial effects of early TCZ administration [27,28]. Severe disease with hypoxemia develops in approximately 15% of COVID-19 patients, usually between 7 and 10 days from the disease beginning [74]. Our data from the tertile analysis of nonlinear variables suggest that the window of opportunity lies between 9 and 12 days from symptom onset—in the early phase of severe disease. This is probably due to the inhibition of immunothrombosis before significant damage occurs, leading to ARDS, as studies in critical COVID-19 requiring mechanical ventilation and ICU transfer report unsatisfactory efficacy in this subset of patients [75,76,77,78]. Moreover, we found that a time period less than or equal to 5 days from the onset of the dyspnea doubled the chance of a good clinical response. Among the three tested hyperinflammatory indices, CCS had predictive value only if the patient did not meet the criteria for a cytokine storm [72]. This is probably due to their attribution to critical patients in CCS and cHIS and basing COV-HI predominantly on the CRP concentration [16,71]. After TCZ administration, non-responders significantly more often met the criteria for COV-HI (41.2% vs. 9.3%), which indicates its potential usefulness in monitoring the response to treatment. 

Age and comorbidities significantly and negatively affect COVID-19 prognosis [79]. In our study, clinical responders had a significantly lower comorbidity burden measured with CCI and were younger than non-responders. Moreover, patients with preexisting atrial fibrillation, chronic coronary syndrome, and obstructive pulmonary diseases were less likely to achieve a good response to TCZ treatment. All abovementioned aspects are established risk factors for poor outcomes in COVID-19 [80,81]. Thus, studies on larger groups are needed to determine whether they independently contribute to worse outcomes after TCZ treatment. As for other clinical predictors, only those linked to either the respiratory rate or function were significant. The ROX index performed slightly better than RR alone and this emphasized its extended clinical utility.

Radiological assessment of the lungs, preferably with chest CT, is essential in patient selection. In our cohort, patients with <70% extent of involved lung parenchyma had six-times greater chances of achieving a good clinical response. Thorough evaluation taking additional radiologic features into consideration, especially those hinting at secondary infections, should be performed. We tested COVID-RRS (a simple tool comprising the affected lung degree, consolidation domination, and presence of pleural effusion) and found that it could perform better in predicting the clinical response than solely the percentage of involved parenchyma [55]. 

LDH activity reflects an excessive inflammatory response, endothelial and pulmonary tissue damage, and hypoxia in COVID-19 and is the best sole laboratory predictor of a critical COVID-19 course and death [7,82,83,84]. We found that initial lower serum activity (<447 U/L) was the strongest laboratory predictor of a good clinical response. It was also the most powerful predictive factor of a good response measured on the second day of TCZ initiation, with values below 438 U/L. In good responders, a slow decrease in LDH activity was observed, whereas non-responders had surging LDH serum activity. Such observations are coherent with the findings of Olewicz-Gawlik et al. and are probably linked to a lesser extent of hyperinflammation, hypoxia-induced cell death, and irreversible organ damage in patients with favorable outcomes [85].

BNP and the hs-TnI serum concentration is an established risk factor for poor outcomes in COVID-19, even in patients without a previous history of heart failure. Their concentrations correlate with myoglobin, D-dimer, procalcitonin, and LDH, proving its role in hyperinflammation and damage assessment [86,87]. A lower hs-TnI (<26 ng/L) concentration was also found to be predictive of a good response, reflecting a lesser extent of cardiac damage [88]. Other significant laboratory indices of low damage and hyperinflammation with predictive value were the BUN concentration <22.2 mg/dL, a moderately low procalcitonin concentration (0.06–0.12 ng/mL), CK serum activity <151 IU/L, and D-dimer levels ≤1.28 µg/mL. BUN and D-dimer levels assessed early after TCZ administration were also predictive of a good response. All the abovementioned biomarkers have been confirmed to influence prognosis in COVID-19 [89,90,91].

Vitamin D deficiency is a proven poor prognostic factor in COVID-19 patients and its supplementation is associated with favorable outcomes, probably due to its immunomodulatory effects [92]. Our study confirmed these findings with a 25(OH)D_3_ serum concentration within the normal range, quadrupling the odds for a good response. This was regardless of the high-dose vitamin D dosing regimen that we instituted in COVID-19 vitamin-D-deficient patients (60,000 IU/week for patients with <10 ng/mL, 40,000 IU/week for patients with 10–30 ng/mL), which can improve SARS-CoV-2 elimination, COVID-19 biomarker levels, and prognosis [93].

Among acute phase reactants, an elevated fibrinogen concentration (≥490 mg/dL) increased the chances of a good clinical response most significantly, confirming the abovementioned arguments of the necessity of early TCZ initiation. An abnormally high concentration of fibrinogen was observed in severe patients and ARDS, whereas a rapid decrease in fibrinogen concentration is associated with a higher mortality risk, consumption coagulopathy, and hepatic dysfunction [94,95]. A higher IL-6 serum concentration (>100 pg/mL) was predictive of TCZ’s effectiveness in COVID-19 [96]. However, markedly rising IL-6, to the degree of >1000 pg/mL, can also be associated with secondary infections and sepsis [14]. In our tertile analysis, we found that a moderately elevated IL-6 (47.4–137.0 pg/mL) serum concentration was associated with a good clinical response. Moreover, a lower IL-6 concentration (<239.3 pg/mL) on the second day after TCZ initiation increased the odds of achieving the primary outcome by nine times. Significant differences in IL-6 levels between non-responders and good responders were also noted on the fifth day after TCZ and in the last reported results. Taking CBC into consideration, only a lower initial leukocyte count (<7.4 G/L) was a predictor of a good clinical response, whereas an unelevated neutrocyte count and associated complex indices (NLR and NMR), immature neutrocyte count, and greater lymphocyte count assessed on the second day after TCZ initiation were linked with a good clinical response.

In RCTs with IL-6 inhibitors, serious adverse events (SAE) were not occurring more frequently in TCZ groups than in comparators (usually including DEX). Most concerns were linked to secondary infections; however, such a risk was not reported in several RCTs and meta-analyses [29,30,32,97,98]. Moreover, some found lower serious infection rates or even fewer severe advent events in patients receiving TCZ added to standard of care (SoC) vs. SoC alone [98,99]. In a study by Naik et al. comparing TCZ monotherapy vs. high-dose DEX, the authors found that the second regimen being SoC was associated with six-times higher mortality and a more than two-times greater secondary infection rate [100]. In our study, 16 (13.3%) patients developed secondary infections after TCZ administration. The majority of secondary infections were mild (UTIs and pneumococcal pneumonia). *Acinetobacter baumanii* VAP was the most serious infectious adverse event and occurred in a third of mechanically ventilated patients. It was followed by bacteremia, sepsis, septic shock, and 100% mortality despite treatment regimens based on colistin [101]. After ARDS, it was the second leading cause of death. Serious outcomes such as MACE, severe lymphopenia, or thrombocytopenia in our study were prevalent upon administration and their onset was less frequently observed after TCZ. Rates of pulmonary embolism and deep vein thrombosis were lower than expected, probably due to the potential antithrombotic effect of immunomodulation and the significant usage of moderately dosed LMWH thromboprophylaxis, as recommended in local guidelines that were updated throughout the pandemic [41]. Adverse events of interest that occurred more commonly after TCZ initiation involved CAC, which was linked to COVID-19 progression and developed significantly more often in non-responders. Others included severe thrombocytopenia, minor bleeding episodes, and kidney and liver injury. The last two were predominantly associated with SARS-CoV-2 and secondary infection, as SLD occurred only in patients with sepsis, and AKI, mostly prerenal, was observed predominantly after ICU transfer. Only one patient in the good responder group met the criteria for DILI linked to TCZ and it was mild, asymptomatic, and self-limiting. Neutropenia was observed both before and after TCZ administration at similar rates, whereas agranulocytosis, mostly transient and clinically irrelevant, was observed more frequently in clinical responders, although the difference was not statistically significant.

Our study has several limitations. We did not study novel biomarkers of COVID-19 hyperinflammation and severity. Moreover, the single-center observational character and limited study population indicate the need for further randomized, multi-center trials with a larger population. This would allow regression analysis and predictive model construction. However, the use of one center study minimized the possibility of bias linked to differences in standards between sites. Predictors described were assessed in patients refractory to DEX. Further studies are needed to establish the prognostic and predictive value of potential clinical, radiological, and laboratory markers in different dosing regimens. We adopted a two-dose regimen regarding previous trials in hyperinflammatory disorders and from our previous experience with severe COVID-19. This may have affected our results. Due to the lack of TCZ initiation as selected by physicians not only on the basis of clinical criteria, but also based on knowledge and local guidelines regarding COVID-19 treatment and thromboprophylaxis, it changed throughout the pandemic and the study. 

Due to reports of beneficial early use and tocilizumab monotherapy, which seems as effective as TCZ+DEX but substantially safer, further studies evaluating the prediction of the response to TCZ without DEX should be tested [100,102,103]. Not only treatment combinations should be evaluated, but also dosing. Considering the noninferiority of baricitinib treatment (to both DEX and TCZ) of severe COVID-19, predictors of other immunomodulatory drugs in COVID-19 should also be thoroughly evaluated [104]. Consequent studies evaluating the efficacy, safety, and optimal dosing regimens of DEX in COVID-19 are also essential for the better tailoring of therapy. The majority of reports come from observational studies, and the results of further RCTs in the field of immunomodulation in COVID-19 are awaited to provide clearer clinical recommendations. SARS-CoV-2 infection, regardless of its severity, can trigger classifiable autoimmune diseases and induces type I interferon (IFN) responses and autoantibody production [1]. Results of long-term studies in the context of long COVID-19 sequelae (including fibroinflammatory interstitial lung disease) with the analysis of COVID-19 severity and instituted treatments are awaited [105]. These could answer questions about whether proper immunomodulation in COVID-19 can reduce the long-term negative outcome risk. Our study also emphasizes the lack of established classification criteria for COVID-19 hyperinflammation, which could predict the response to immunomodulatory therapy and be also useful in therapy monitoring. Despite the remarkably low thrombotic adverse event rates, further studies need to consider the immunomodulatory effect on endothelitis and thrombosis in severe COVID-19. Deepening the knowledge of established biomarkers’ utility is needed, such as the optimization of IL-6 cut-offs in patient selection for immunomodulatory treatment. Novel biomarkers in COVID-19 reflecting endothelial damage (i.e., intercellular adhesion molecule 1, ICAM1), inflammation (i.e., interleukin-1 and -6 receptors, IL-1R and IL-6R; interleukin-8 and -18, IL-8 and IL-18; soluble Fas ligand, sFasL; granulocyte–macrophage colony-stimulating factor, GM-CSF; and soluble urokinase plasminogen activator receptor, suPAR), immune dysregulation (autoantibodies against tissues, cytokines, and chemokines, including autoantibodies against type I interferon, antiphospholipid antibodies, antinuclear antibodies), or genetic risk (*TYK2*, *IFNAR2*, *CCR2*, and others) should be studied [106,107,108].

## 5. Conclusions

Immunomodulatory therapy in severe COVID-19 should be individualized by taking predictive outcomes into consideration. Adding TCZ in severe COVID-19 patients not improving despite dexamethasone treatment is most beneficial in the early phase of hyperinflammation (between 9 and 12 days from symptom onset). It is more effective in patients with better respiratory function and with lower hyperinflammation and tissue damage laboratory indices. We identified forty-nine clinical, laboratory, and radiological predictive factors of tocilizumab treatment in severe COVID-19 not improving despite dexamethasone. A time from symptom onset between 9 and 12 days, involvement of less than 70% of lung parenchyma in chest CT, and lower activity of lactate dehydrogenase were the strongest. They can affect the optimization of patient selection and treatment monitoring in the immunomodulatory treatment of SARS-CoV-2 complications.

## Figures and Tables

**Table 1:** Basic demographic and initial clinical characteristics of study group, presented as n (%), mean (±SD), or median (Q1–Q3).

Characteristic	Clinical Responders	Non-Responders	*p*-Value
Patients	86 (71.7%)	34 (28.3%)	-
Sex, male	59 (68.6%)	19 (55.9%)	0.188 ^a^
Age (years) *	56.1 (±13.2)	63.5 (±12.5)	0.006 ^b^
Body mass index (kg/m^2^)	27.8 (24.7–32.3)	30.6 (26.1–33.2)	0.157 ^c^
Charlson Comorbidity Index *	2 (1–3)	3 (2–4)	0.004 ^c^
Comorbidities:			
atrial fibrillation *	5 (5.8%)	7 (20.6%)	0.024 ^d^
chronic coronary syndrome *	9 (10.5%)	10 (29.4%)	0.010 ^d^
asthma and/or COPD *	4 (4.7%)	6 (17.7%)	0.029 ^d^
Time from symptom onset to admission (days)	11 (8–12)	9 (8–13)	0.315 ^c^
Time from dyspnea onset to admission (days)	2 (0–4)	2 (1–4)	0.136 ^c^
Duration of hospitalization (days)	13 (11–17)	14 (10–22)	0.553 ^c^
Time from symptom onset to TCZ (days)	12 (9–13)	10 (9–14)	0.337 ^c^
Time from dyspnea onset to TCZ (days)	3 (2–6)	4.5 (3–7)	0.086 ^c^
Lung parenchyma involvement in CT (%) *	50 (35–60)	70 (60–85)	<0.001 ^c^
COVID-RRS *	5.0 (4.0–6.0)	7.3 (5.6–8.5)	<0.001 ^c^
Baseline PaO_2_/FiO_2_ *	203 (136–277)	106 (80–177)	<0.001 ^c^
SpO_2_/FiO_2_ on TCZ initiation *	179 (120–239)	111 (102–152)	<0.001 ^c^
WHO clinical progression scale on TCZ initiation *			
5	75 (87.2%)	23 (67.6%)	0.013 ^a^
6	11 (12.7%)	11 (32.4%)	
Concomitant treatment:			
remdesivir	71 (82.6%)	27 (79.4%)	0.688 ^a^
convalescent plasma	61 (70.9%)	24 (70.5%)	0.970 ^a^
LMWH	84 (97.7%)	32 (94.1%)	0.318 ^d^

* *p* < 0.05—^a^ χ^2^ test, ^b^ *t*-Student test, ^c^ Mann–Whitney U test or ^d^ Fisher’s exact test, as appropriate COPD—chronic obstructive pulmonary disease, TCZ—tocilizumab, CT—computed tomography, COVID-RRS—COVID-19 Radiological Risk Score, PaO_2_—partial pressure of arterial oxygen, FiO_2_—fraction of inspired oxygen, SpO_2_—peripheral oxygen saturation measured with pulse oximeter, WHO—World Health Organization, LMWH—low-molecular-weight heparin.

**Table 2:** Clinical and radiological predictors of clinical response before tocilizumab treatment of severe COVID-19 identified in univariate logistic regression analysis on adjusted data.

Predictor	OR	95%CI	*p* Value
COVID-RRS ≤ 6.5	7.37	3.06–17.79	<0.001
<70% of lung parenchyma involvement	6.76	2.63–17.36	<0.001
9–12 days from symptom onset (vs. <9 and >12)	6.43	1.82–22.73	0.004
ROX index ≥ 8.51	5.77	2.35–14.27	<0.001
RR < 20 breaths/min	5.40	2.29–12.75	<0.001
No indices of AMI	5.39	1.87–15.51	0.002
HR-ROX index ≥ 11.59	4.97	2.02–12.59	<0.001
No CCS on administration day	4.70	1.81–12.23	0.001
qSOFA = 0	4.55	2.03–10.22	<0.001
SpO_2_/FiO_2_ > 122	4.47	1.92–10.40	<0.001
cHIS score < 3	4.44	1.43–13.77	0.009
Lack of asthma/COPD	4.39	1.55–16.71	0.030
Lack of atrial fibrillation	4.20	1.23–14.33	0.022
Baseline PaO_2_/FiO_2_ > 200 mmHg	4.04	1.59–10.27	0.003
Age < 65 years	3.69	1.60–8.46	0.002
Lack of ischemic heart disease	3.56	1.29–9.79	0.014
No HFNOT (WHO Ordinal Scale = 5)	3.26	1.25–8.49	0.016
CCI < 4	2.96	1.31–6.71	0.009
≤5 days from onset of dyspnea	2.43	1.06–5.56	0.035

OR—odds ratio, 95%CI—95% confidence interval, COVID-RRS—COVID-19 Radiological Risk Score, RR—respiratory rate, AMI—acute myocardial injury, CCS—COVID-19 cytokine storm, qSOFA—Quick Sepsis-Related Organ Failure Assessment score, SpO_2_—peripheral blood saturation measured with finger pulse oximeter, FiO_2_—fraction of inspired oxygen, cHIS—COVID-19-associated Hyperinflammation Syndrome score, COPD—chronic obstructive pulmonary disease, PaO_2_—partial pressure of arterial oxygen, HFNOT—high-flow nasal cannula oxygen therapy, WHO—World Health Organization, CCI—Charlson Comorbidity Index.

**Table 3:** Laboratory predictors of good response (measured in tocilizumab administration day). Analyses have been conducted on adjusted data.

Predictor	OR	95%CI	*p*-Value
LDH < 447 U/L	12.67	4.42–36.31	<0.001
BNP < 50.5 pg/mL	6.57	2.73–15.83	<0.001
hs-TnI < 26 ng/L	4.80	1.55–14.81	0.006
fibrinogen ≥ 490 mg/dL	4.46	1.86–10.72	<0.001
BUN < 22.2 mg/dL	4.17	2.02–10.99	0.017
PCT 0.06–0.12 ng/mL	3.98	1.40–11.28	0.009
25(OH)D_3_ ≥ 30 ng/mL	3.20	1.20–8.54	0.020
D-Dimer ≤ 1.28 µg/mL	3.12	1.37–7.09	0.006
IL-6 47.4–137.0 pg/mL	3.07	1.90–4.98	<0.001
WBC < 7.4 G/L	2.74	1.20–6.25	0.017
CK < 151 IU/L	2.62	1.16–5.91	0.020
lack of pyuria	2.5	1.04–5.31	0.039

OR—odds ratio, 95%CI—95% confidence interval, LDH—lactate dehydrogenase, hs-TnI—high sensitive troponin I, BUN—blood urea nitrogen, 25(OH)D_3_—calcifediol, PCT—procalcitonin, IL-6—interleukin 6, WBC—white blood cell count, CK—creatinine kinase.

**Table 4:** Clinical and laboratory early predictors of good response (on 2nd day after TCZ) identified in univariate logistic regression analysis on adjusted data.

Predictor	OR	95%CI	*p* Value
LDH < 439 IU/L	46.55	10.30–210.4	<0.001
QO_2_ < 14 L/min	19.83	7.23–54.43	<0.001
SpO_2_/FiO_2_ >132	17.63	6.34–49.06	<0.001
WHO Ordinal Scale < 6	13.83	5.20–36.73	<0.001
ROX index > 8.61	13.60	5.13–36.06	<0.001
MEWS < 2	12.79	4.14–39.54	<0.001
HR-ROX index > 12.47	12.73	4.82–33.59	<0.001
Neutrocytes < 4.8 G/L	9.17	3.41–24.65	<0.001
IL-6 < 239.3 pg/mL	9.17	3.41–24.65	<0.001
NLR < 5.5	9.07	3.68–22.35	<0.001
RR < 17/min	7.92	3.24–19.31	<0.001
D-dimer < 4.40 µg/mL	7.81	3.14–19.43	<0.001
NMR < 12.1	6.87	2.84–16.62	<0.001
qSOFA = 0	6.56	2.74–15.68	<0.001
WBC < 7.1 G/L	4.93	2.04–11.88	<0.001
BUN < 20.2 mg/dL	3.80	1.65–8.76	0.002
Lymphocytes > 1.30 G/L	3.20	1.20–8.54	0.016
INC < 0.07 G/L	2.80	1.23–6.40	0.013

OR—odds ratio, 95%CI—95% confidence interval, LDH—lactate dehydrogenase, QO_2_—oxygen flow, SpO_2_—peripheral blood saturation measured with finger pulse oximeter, FiO_2_—fraction of inspired oxygen, WHO—World Health Organization, MEWS—Modified Early Warning Score, IL-6—interleukin 6, NLR—neutrocyte-to-lymphocyte ratio, RR—respiratory rate, NMR—neutrocyte-to-monocyte ratio, qSOFA—Quick Sepsis-Related Organ Failure Assessment score, WBC—white blood cell count, BUN—blood urea nitrogen, INC—immature neutrocyte count.

**Table 5:** Mortality predictors (assessed on the day of tocilizumab initiation, apart from two last predictors, which were measured during admission) and their comparison to prediction of good clinical response. Cut-offs are listed as linear predictors and range is presented for nonlinear associations. Analyses have been conducted on adjusted data.

Predictor	Mortality			Good Response		
	OR (95%CI)	Cut-Off/Range	*p* Value	OR (95%CI)	Cut-Off/Range	*p*-Value
RR (/min)	10.7 (3.3–34.4)	>2	<0.001	5.4 (2.3–12.8)	<20	<0.001
ROX	10.0 (2.77–36.09)	<8.74	<0.001	5.8 (2.4–14.3)	≥8.51	0.004
HR-ROX	8.8 (2.4–31.7)	<12.6	<0.001	5.0 (2.0–12.6)	≥11.59	<0.001
qSOFA	8.9 (2.8–28.8)	≥1	<0.001	4.6 (2.0–10.2)	=0	<0.001
WHO OS	7.3 (2.5–21.2)	>5	<0.001	3.3 (1.3–8.5)	=5	0.016
SpO_2_/FiO_2_	26.8 (7.7–92.6)	<111	<0.001	4.5 (1.9–10.4)	>122	0.068
age (years)	4.9 (1.6–14.6)	>62	0.002	3.7 (1.6–8.5)	<65	0.002
CCI	8.5 (2.6–27.5)	≥3.0		3.0 (1.3–6.7)	<4	0.009
days from symptom onset	NS	-	0.499	6.4 (1.8–22.7)	9–12	0.004
days from dyspnea onset	NS	-	0.124	2.4 (1.1–5.6)	≤5	0.035
COVID-RRS	17.0 (4.6–62.9)	≥6.5	< 0.001	6.8 (1.8–22.7)	≤6.5	<0.001
lung involvement (%)	12.5 (4.4–36.1)	≥70	<0.001	7.4 (3.1–17.8)	<70	<0.001
WBC (G/L)	14.9 (1.9–115.9)	>5.2	<0.001	13 (15.1%)	<7.4	0.017
NLR	6.3 (2.0–20.1)	>6.2	0.001	NS	-	0.056
SCr (mg/dL)	6.3 (1.4–28.4)	≥0.8	0.009	NS	-	0.098
BUN (mg/dL)	11.1 (3.7–33.3)	>22.2	<0.001	4.2 (2.0–11.0)	<22.2	0.017
D-dimer (µg/mL)	4.7 (1.6–13.9)	>1.11	0.006	3.1 (1.4–7.1)	≤1.28	0.006
fibrinogen (mg/dL)	3.6 (1.3–9.9)	<479	0.014	4.5 (1.9–10.7)	≥490	<0.001
IL-6 (pg/mL)	NS	-	0.573	3.1 (1.9–5.0)	47.4–137.0	<0.001
LDH (U/L)	18.2 (5.4–61.0)	≥530	<0.001	12.7 (4.4–36.3)	<447	<0.001
CK (IU/L)	4.4 (1.4–13.3)	>308	0.009	2.6 (1.2–5.9)	<151	0.020
BNP (pg/mL)	5.0 (1.7–14.1)	>51.20	0.002	6.6 (2.7–15.8)	<50.5	<0.001
PCT (ng/mL)	3.2 (1.2–8.5)	>0.13	0.004	4.0 (1.4–11.3)	0.06–0.12	0.009
hs-TnI (ng/L)	6.2 (1.9–19.9)	>26	0.004	4.8 (1.6–14.8)	<26	0.006
baseline PaO_2_/FiO_2_ (mmHg)	10.5 (3.6–30.7)	<100	<0.001	4.0 (1.6–10.3)	>200	0.003
baseline 25(OH)D_3_ (ng/mL)	6.9 (1.9–25.1)	<27	0.003	3.2 (1.2–8.5)	≥30	0.009

OR—odds ratio, 95%CI—95% confidence interval, NS—nonsignificant, RR—respiratory rate, qSOFA—Quick Sepsis-Related Organ Failure Assessment score, WHO OS—World Health Organization Ordinal Scale, SpO_2_—peripheral blood saturation measured with finger pulse oximeter, FiO_2_—fraction of inspired oxygen, CCI—Charlson Comorbidity Index, COVID-RRS—COVID-19 Radiological Risk Score, WBC—white blood cell count, NLR—neutrocyte-to-lymphocyte ratio, SCr—serum creatinine, BUN—blood urea nitrogen, IL-6—interleukin-6, LDH—lactate dehydrogenase, CK—creatinine kinase, BNP—B-type natriuretic peptide, PCT—procalcitonin, hs-TnI—high specific troponin I, 25(OH)D_3_—calcifediol.

**Table 6:** Major outcomes and their prevalence upon administration and occurrence after treatment with tocilizumab in studied groups. Analyses have been conducted on adjusted data.

Outcome	Prevalence upon Administration			Occurence after Administration		
	Clinical Responders	Non-Responders	*p*-Value	Clinical Responders	Non-Responders	*p*-Value
death *	-	-	-	-	20 (58.8%)	<0.001 ^a^
ICU transfer due to ARDS progression *	-	-	-	-	20 (58.8%)	<0.001 ^a^
CAC *	-	2 (5.9%)	0.078 ^b^	31 (36.1%)	22 (64.7%)	0.004 ^a^
neutropenia	15 (17.4%)	3 (8.8%)	0.233 ^a^	13 (15.1%)	2 (5.9%)	0.140 ^b^
agranulocytosis	-	-	-	10 (11.6%)	2 (5.9%)	0.282 ^b^
major bleeding	-	-	-	-	1 (2.9%)	0.283 ^b^
MACE	8 (9.3%)	11 (32.4%)	0.001 ^a^	3 (3.5%)	9 (26.5%)	<0.001 ^b^
liver injury	28 (32.6%)	9 (26.5%)	0.515 ^a^	18 (20.9%)	15 (44.1%)	0.010 ^a^
DILI	-	-	-	1 (1.2%)	-	0.716 ^b^
patients with secondary infections	2 (2.3%)	4 (11.8%)	0.033 ^a^	6 (7.0%)	10 (29.4%)	0.002 ^b^
AKI	-	-	-	6 (7.0%)	17 (50%)	<0.001 ^a^
acute tubulointerstitial nephritis	6 (7.0%)	-	0.182 ^b^	4 (4.7%)	2 (5.9%)	0.547 ^b^
hemodynamic instability	-	-	-	6 (7.0%)	3 (8.8%)	0.711 ^b^
respiratory deterioration	-	-	-	13 (15.1%)	26 (76.5%)	<0.001 ^a^
COV-HI	50 (58.1%)	23 (67.7%)	0.336 ^a^	8 (9.3%)	14 (41.2%)	<0.001 ^a^
cHIS	65 (75.6%)	30 (88.3%)	0.124 ^a^	4 (4.7%)	3 (8.8%)	0.313 ^b^
CCS	10 (11.6%)	13 (38,2%)	<0.001 ^a^	4 (4.7%)	3 (8.8%)	0.313 ^b^

* *p* < 0.05—^a^ χ^2^ test or ^b^ Fisher’s exact test; ICU—intensive care unit, CAC—COVID-19-associated coagulopathy, MACE—major adverse cardiovascular event, DILI—drug-induced liver injury, AKI—acute kidney injury, COV-HI—COVID-19 hyperinflammation syndrome, cHIS—COVID-19-Associated Hyperinflammation Syndrome score, CCS—COVID-19 cytokine storm.

## Data Availability

The data presented in this study are available on reasonable request from the corresponding author. The data are not publicly available due to a lack of patients’ consent to public data sharing.

## Supplementary Materials

The following supporting information can be downloaded at: https://www.mdpi.com/article/10.3390/jcm12062429/s1, Table S1. Definitions of additional outcomes of the study. Table S2. Full demographic and initial clinical characteristics of study group, presented as n (%), mean (±SD) or median (Q1–Q3). Table S3. Extended clinical characteristics of study groups, presented as median (Q1–Q3). *p* values were derived with Mann-Whitney U test. Table S4. Extended laboratory characteristics of studied groups and outcomes, presented as median (Q1–Q3). *p* values were derived with Mann-Whitney U test. Table S5. Outcomes and their prevalence upon administration and occurrence after treatment with tocilizumab in studied groups.
