# Supplementary material for: Identification of Clinical Response Predictors of Tocilizumab Treatment in Patients with Severe COVID-19 Based on Single-Center Experience
Source: J Clin Med. 2023 Mar 22;12(6):2429. doi: 10.3390/jcm12062429 (PMC10051490; doi:10.3390/jcm12062429)
Supplement: Supplementary file 1 [file jcm-12-02429-s001.zip › Table S1.pdf]

Journal of
Clinical Medicine

Supplementary Table S1. Definitions of additional outcomes of the study.

Outcome	Description and/or reference
Acute Respiratory Distress Syndrome (ARDS)	Diagnosed and stratified by Berlin definition [1].
Deep vein thrombosis (DVT)	Diagnosed per European Society of Cardiology (ESC) recommendations [2]
Pulmonary embolism (PE)	Diagnosed in accordance with European Society of Cardiology (ESC) recommendations [3]
COVID-19-associated coagulopathy (CAC)	Diagnosed upon the presence of two or more of the following criteria by Iba et al. [4]:<br>• low platelet count,<br>• elevated D-dimer concentration,<br>• prolonged prothrombin count,<br>• clinical presence of macro- and/or microthrombosis.
Disseminated intravascular coagulation (DIC)	Diagnosed and scored as recommended by the International Society on Thrombosis and Haemostasis (ISTH) [5,6]
Severe lymphopenia	Defined as lymphocyte count < 0.6 $*10^3/\mu L$
Hemolytic anemia	Anemia with positive direct antiglobulin test (DAT), elevated reticulocyte count, elevated indirect bilirubin, and decreased haptoglobin serum concentration
Neutropenia	Absolute neutrocyte count (ANC) < 1.0 $*10^3/\mu L$
Agranulocytosis	Absolute neutrocyte count (ANC) < 0.5 $*10^3/\mu L$
Thrombocytopenia	platelet (PLT) count < 150 $*10^3/\mu L$
Severe thrombocytopenia	Platelet (PLT) count < 50 $*10^3/\mu L$
Major bleeding episode	Defined by the International Society on Thrombosis and Haemostasis (ISTH) in non-surgical patients [7]
Minor bleeding episode	Bleeding episode not fulfilling the criteria of a major bleeding episode as defined by ISTH [7]
Major adverse cardiovascular events (MACE)	The modified definition used by Nauffal et al. [8]: a composite of venous thromboembolism, myocardial infarction or myocardial injury, ischemic stroke, transient ischemic attack, systemic embolism, major adverse limb events, heart

	failure decompensation or heart failure *de novo*, new atrial fibrillation and myocarditis.
Acute myocardial injury (AMI) and acute coronary syndromes (ACS)	Based on definitions provided in the Fourth Universal Definition of Myocardial Infarction [9]
Myocarditis	Diagnosed with the proposed by ESC definition of clinically suspected myocarditis [10]
Hypotension	Mean blood pressure (MBP) of 70 mmHg and less
Hemodynamic instability	Hypotension refractory to fluid therapy requiring intravenous catecholamines to achieve MAP greater than 70 mmHg
Liver injury and its type	Abnormal liver enzyme activity in serum (elevated at least two times above normal range). Types: hepatocellular, cholestatic, and mixed were defined in the American College of Gastroenterology Clinical Guideline [11]
Drug-induced liver injury (DILI)	Any one of the following, as defined by Aithal et al. [12]:<br>(a) elevation ≥ 5 x above the upper limit of normal (ULN) of alanine aminotransferase (ALT) serum activity<br>(b) elevation ≥ 2 x above the upper limit of normal (ULN) of alkaline phosphatase (ALP) activity (particularly with accompanying peaks in the activity of gamma-glutamyltranspeptidase (GGT) in the absence of known bone pathology driving the rise in ALP level)<br>(c) elevation ≥ 3 x above the upper limit of normal (ULN) of alanine aminotransferase (ALT) serum activity and simultaneous elevation of total bilirubin concentration > 2 x ULN<br>and at least a probable causal relationship with the drug defined as the Roussel Uclaf Causality Assessment Method (RUCAM) score of at least 6 [13]
Severe liver dysfunction (SLD)	Total bilirubin ≥ 2 mg/dl or elevation of aminotransferase levels (> 20-fold ULN), as defined by Roedl et al. [14]
Sepsis and septic shock	Diagnosed and stratified per The Third International Consensus Definitions for Sepsis and Septic Shock (Sepsis-3) [15]
Acute kidney injury (AKI)	Definition and severity assessment based on The Kidney Disease: Improving Global Outcomes (KDIGO) guidelines [16]
Proteinuria	Significant proteinuria was defined in urine protein concentration (UPC) in urinalysis ≥ 500 mg/dL

Sterile pyuria	Presence of elevated leucocyte counts in urinalysis with negative results for urine culture
Acute tubulointerstitial nephritis (ATN)	Clinical diagnosis made on laboratory markers of kidney tubular injury: significant proteinuria with sterile pyuria (both defined above) with or without erythrocyturia and/or urinary casts and/or elevation in kidney function parameters [17–19]
Acute confusional state	Diagnosed with Diagnostic and Statistical Manual of Mental Disorders, 5th edition (DSM-V) delirium criteria [20]
COVID-19 encephalopathy	Encephalopathy in severe COVID-19 patients after exclusion of other potential causes with good response to glucocoricoid treatment, adapted from Michael et al [21]
COVID-19 hyperinflammation syndrome (COV-HI)	Defined by Manson et al. as [22]:<br>(1) C-reactive protein (CRP) concentration greater than 150 mg/L, or<br>(2) Doubling CRP concentration within 24 h from greater than 50 mg/L, or<br>(3) Ferritin concentration greater than 1500 μg/L
COVID-19-associated hyperinflammation syndrome (cHIS) and cHIS score	As proposed by Manson et al [23] – cHIS score of 3 or more (presence of 3 or more simultaneously):<br>1) Fever defined as a temperature of ⩾38.0°C<br>2) Macrophage activation defined as serum ferritin concentration ≥700 μg/L<br>3) Hematological dysfunction defined as NLR (neutrocyte-to-lymphocyte) ≥10 OR both hemoglobin concentration ≤9.2 g/dL and platelet count ≤110 × 10^9/ L<br>4) Coagulopathy defined as a D-dimer concentration ≥1.5 μg/mL<br>5) Hepatic injury defined as a lactate dehydrogenase (LDH) concentration ≥400 U/L, or an aspartate aminotransferase (AST) concentration ≥100 U/L<br>6) Cytokinaemia defined as an interleukin-6 (IL-6) serum concentration ≥15 pg/mL, or a triglyceride concentration ≥150 mg/dL, or CRP concentration ≥150 mg/L
COVID-19 cytokine storm (CCS)	Classified upon meeting the Temple criteria [24]. Patients needed to meet all the entry criteria and at least one criterion per cluster. Entry criteria consisted of: signs/symptoms of COVID-19, RT-PCR positive for COVID-19, ground glass opacities in chest computed tomography (CT), serum ferritin >250 ng/mL and CRP >46 mg/L. Clusters included:<br>(1) Cluster I – inflammatory parameters (albumin <28 g/L, percentage of lymphocytes in complete blood count (CBC) <10.2% or absolute neutrocyte count > 11.4 G/L)<br>(2) Cluster II - cell death and tissue damage biomarkers (ALT >60 U/L, AST >87 U/L, D-dimers > 4.98 μg/mL, LDH >416 U/L or troponin I >1.09 ng/mL)

(3) Cluster III - prerenal electrolyte imbalance indices (anion gap <6.8 mmol/L, serum chloride >106 mmol/L, serum potassium >4.9 mmol/:, BUN/creatinine ratio >29.

1. The ARDS Definition Task Force* Acute Respiratory Distress Syndrome: The Berlin Definition. *JAMA* **2012**, *307*, 2526–2533, doi:10.1001/jama.2012.5669.
2. Mazzolai, L.; Aboyans, V.; Ageno, W.; Agnelli, G.; Alatri, A.; Bauersachs, R.; Brekelmans, M.P.A.; Büller, H.R.; Elias, A.; Farge, D.; et al. Diagnosis and Management of Acute Deep Vein Thrombosis: A Joint Consensus Document from the European Society of Cardiology Working Groups of Aorta and Peripheral Vascular Diseases and Pulmonary Circulation and Right Ventricular Function. *Eur Heart J* **2018**, *39*, 4208–4218, doi:10.1093/eurheartj/ehx003.
3. Konstantinides, S.V.; Meyer, G.; Becattini, C.; Bueno, H.; Geersing, G.-J.; Harjola, V.-P.; Huisman, M.V.; Humbert, M.; Jennings, C.S.; Jiménez, D.; et al. 2019 ESC Guidelines for the Diagnosis and Management of Acute Pulmonary Embolism Developed in Collaboration with the European Respiratory Society (ERS). *Eur Heart J* **2020**, *41*, 543–603, doi:10.1093/eurheartj/ehz405.
4. Iba, T.; Warkentin, T.E.; Thachil, J.; Levi, M.; Levy, J.H. Proposal of the Definition for COVID-19-Associated Coagulopathy. *Journal of Clinical Medicine* **2021**, *10*, 191, doi:10.3390/jcm10020191.
5. Taylor, F.B.; Toh, C.H.; Hoots, W.K.; Wada, H.; Levi, M.; Scientific Subcommittee on Disseminated Intravascular Coagulation (DIC) of the International Society on Thrombosis and Haemostasis (ISTH) Towards Definition, Clinical and Laboratory Criteria, and a Scoring System for Disseminated Intravascular Coagulation. *Thromb Haemost* **2001**, *86*, 1327–1330.
6. Toh, C.H.; Hoots, W.K.; SSC on Disseminated Intravascular Coagulation of the ISTH The Scoring System of the Scientific and Standardisation Committee on Disseminated Intravascular Coagulation of the International Society on Thrombosis and Haemostasis: A 5-Year Overview. *J Thromb Haemost* **2007**, *5*, 604–606, doi:10.1111/j.1538-7836.2007.02313.x.
7. Schulman, S.; Kearon, C.; Subcommittee on Control of Anticoagulation of the Scientific and Standardization Committee of the International Society on Thrombosis and Haemostasis Definition of Major Bleeding in Clinical Investigations of Antihemostatic Medicinal Products in Non-Surgical Patients. *J Thromb Haemost* **2005**, *3*, 692–694, doi:10.1111/j.1538-7836.2005.01204.x.
8. Nauffal, V.; Achanta, A.; Goldhaber, S.Z.; Piazza, G. Association of ABO Blood Group Type with Cardiovascular Events in COVID-19. *J Thromb Thrombolysis* **2021**, doi:10.1007/s11239-020-02364-5.
9. Thygesen, K.; Alpert, J.S.; Jaffe, A.S.; Chaitman, B.R.; Bax, J.J.; Morrow, D.A.; White, H.D.; Executive Group on behalf of the Joint European Society of Cardiology (ESC)/American College of Cardiology (ACC)/American Heart Association (AHA)/World Heart Federation (WHF) Task Force for the Universal Definition of Myocardial Infarction Fourth Universal Definition of Myocardial Infarction (2018). *J Am Coll Cardiol* **2018**, *72*, 2231–2264, doi:10.1016/j.jacc.2018.08.1038.
10. Caforio, A.L.P.; Pankuweit, S.; Arbustini, E.; Basso, C.; Gimeno-Blanes, J.; Felix, S.B.; Fu, M.; Heliö, T.; Heymans, S.; Jahns, R.; et al. Current State of Knowledge on Aetiology, Diagnosis, Management, and Therapy of Myocarditis: A Position Statement of the European Society of Cardiology Working Group on Myocardial and Pericardial Diseases. *Eur Heart J* **2013**, *34*, 2636–2648, 2648a–2648d, doi:10.1093/eurheartj/eht210.
11. Kwo, P.Y.; Cohen, S.M.; Lim, J.K. ACG Clinical Guideline: Evaluation of Abnormal Liver Chemistries. *Official journal of the American College of Gastroenterology | ACG* **2017**, *112*, 18–35, doi:10.1038/ajg.2016.517.
12. Aithal, G.P.; Watkins, P.B.; Andrade, R.J.; Larrey, D.; Molokhia, M.; Takikawa, H.; Hunt, C.M.; Wilke, R.A.; Avigan, M.; Kaplowitz, N.; et al. Case Definition and Phenotype Standardization in Drug-Induced Liver Injury. *Clinical Pharmacology & Therapeutics* **2011**, *89*, 806–815, doi:https://doi.org/10.1038/clpt.2011.58.
13. Roussel Uclaf Causality Assessment Method (RUCAM) in Drug Induced Liver Injury. In *LiverTox: Clinical and Research Information on Drug-Induced Liver Injury*; National Institute of Diabetes and Digestive and Kidney Diseases: Bethesda (MD), 2012.

14. Roedl, K.; Jarczak, D.; Drolz, A.; Wichmann, D.; Boenisch, O.; de Heer, G.; Burdelski, C.; Frings, D.; Sensen, B.; Nierhaus, A.; et al. Severe Liver Dysfunction Complicating Course of COVID-19 in the Critically Ill: Multifactorial Cause or Direct Viral Effect? *Annals of Intensive Care* **2021**, *11*, 44, doi:10.1186/s13613-021-00835-3.
15. Singer, M.; Deutschman, C.S.; Seymour, C.W.; Shankar-Hari, M.; Annane, D.; Bauer, M.; Bellomo, R.; Bernard, G.R.; Chiche, J.-D.; Coopersmith, C.M.; et al. The Third International Consensus Definitions for Sepsis and Septic Shock (Sepsis-3). *JAMA* **2016**, *315*, 801–810, doi:10.1001/jama.2016.0287.
16. Khwaja, A. KDIGO Clinical Practice Guidelines for Acute Kidney Injury. *Nephron Clin Pract* **2012**, *120*, c179-184, doi:10.1159/000339789.
17. Ruebner, R.L.; Fadrowski, J.J. Tubulointerstitial Nephritis. *Pediatric Clinics of North America* **2019**, *66*, 111–119, doi:10.1016/j.pcl.2018.08.009.
18. Sharma, P.; Uppal, N.N.; Wanchoo, R.; Shah, H.H.; Yang, Y.; Parikh, R.; Khanin, Y.; Madireddy, V.; Larsen, C.P.; Jhaveri, K.D.; et al. COVID-19-Associated Kidney Injury: A Case Series of Kidney Biopsy Findings. *J Am Soc Nephrol* **2020**, *31*, 1948–1958, doi:10.1681/ASN.2020050699.
19. Kudose, S.; Batal, I.; Santoriello, D.; Xu, K.; Barasch, J.; Peleg, Y.; Canetta, P.; Ratner, L.E.; Marasa, M.; Gharavi, A.G.; et al. Kidney Biopsy Findings in Patients with COVID-19. *J Am Soc Nephrol* **2020**, *31*, 1959–1968, doi:10.1681/ASN.2020060802.
20. European Delirium Association; American Delirium Society The DSM-5 Criteria, Level of Arousal and Delirium Diagnosis: Inclusiveness Is Safer. *BMC Med* **2014**, *12*, 141, doi:10.1186/s12916-014-0141-2.
21. Michael, B.D.; Walton, D.; Westenberg, E.; García-Azorín, D.; Singh, B.; Tamborska, A.A.; Netravathi, M.; Chomba, M.; Wood, G.K.; Easton, A.; et al. Consensus Clinical Guidance for Diagnosis and Management of Adult COVID-19 Encephalopathy Patients. *JNP* **2022**, appi.neuropsych.22010002, doi:10.1176/appi.neuropsych.22010002.
22. Manson, J.J.; Crooks, C.; Naja, M.; Ledlie, A.; Goulden, B.; Liddle, T.; Khan, E.; Mehta, P.; Martin-Gutierrez, L.; Waddington, K.E.; et al. COVID-19-Associated Hyperinflammation and Escalation of Patient Care: A Retrospective Longitudinal Cohort Study. *The Lancet Rheumatology* **2020**, *2*, e594–e602, doi:10.1016/S2665-9913(20)30275-7.
23. Webb, B.J.; Peltan, I.D.; Jensen, P.; Hoda, D.; Hunter, B.; Silver, A.; Starr, N.; Buckel, W.; Grisel, N.; Hummel, E.; et al. Clinical Criteria for COVID-19-Associated Hyperinflammatory Syndrome: A Cohort Study. *Lancet Rheumatol* **2020**, *2*, e754–e763, doi:10.1016/S2665-9913(20)30343-X.
24. Caricchio, R.; Gallucci, M.; Dass, C.; Zhang, X.; Gallucci, S.; Fleece, D.; Bromberg, M.; Criner, G.J.; Temple University COVID-19 Research Group Preliminary Predictive Criteria for COVID-19 Cytokine Storm. *Ann Rheum Dis* **2021**, *80*, 88–95, doi:10.1136/annrheumdis-2020-218323.
