# Supplementary material for: Identification of Clinical Response Predictors of Tocilizumab Treatment in Patients with Severe COVID-19 Based on Single-Center Experience
Source: J Clin Med. 2023 Mar 22;12(6):2429. doi: 10.3390/jcm12062429 (PMC10051490; doi:10.3390/jcm12062429)
Supplement: Supplementary file 1 [file jcm-12-02429-s001.zip › Table S2.pdf]

Journal of
Clinical Medicine

MDPI

Supplementary Table S2. Full demographic and initial clinical characteristics of study group, presented as n (%), mean (±SD) or median (Q1-Q3).

Characteristic	Clinical responders	Non-responders	*P* value
Patients	86 (71.7%)	34 (28.3%)	-
Sex, male	59 (68.6%)	19 (55.9%)	0.188[a]
Age (years)*	56.1 (±13.2)	63,5 (±12.5)	0.006[b]
Vaccinated against SARS-CoV-2	3 (3.5%)	2 (5.9%)	0.438[d]
SARS-CoV-2 reinfection	1 (1.1%)	1 (2.9%)	0.488[d]
Body Mass Index (kg/m^2)	27.8 (24.7-32.3)	30.6 (26.1-33.2)	0.157[c]
Charleson Comorbidity Index*	2 (1-3)	3 (2-4)	0.004[c]
Comorbidities:			
obesity	32 (37.2%)	17 (50.0%)	0.199[a]
arterial hypertension	40 (46.5%)	19 (55.9%)	0.354[a]
hyperlipidaemia	31 (36.0%)	17 (50.0%)	0.159[a]
atrial fibrillation*	5 (5.8%)	7 (20.6%)	0.024[d]
chronic kidney disease	4 (4.7%)	3 (8.8%)	0.313[d]
chronic coronary syndrome*	9 (10.5%)	10 (29.4%)	0.010[d]
chronic heart failure	3 (3.5%)	3 (8.8%)	0.22[d]
diabetes mellitus	19 (22.1%)	9 (26.5%)	0.609 [a]
connective tissue disorders	7 (8.1%)	3 (8.8%)	0.578[d]
inflammatory bowel disease	2 (2.3%)	-	0.512[d]
transplant recipients	3 (3.5%)	-	0.364[d]
asthma and/or COPD*	4 (4.7%)	6 (17.7%)	0.029[d]
active neoplastic disease	3 (3.5%)	1 (2.9%)	0.682[d]
chronic hepatic disorder	1 (1.1%)	1 (2.9%)	0.488[d]
Time from symptoms onset to admission (days)	11 (8-12)	9 (8-13)	0.315[c]
Time from dyspnoea onset to admission (days)	2 (0-4)	2 (1-4)	0.136[c]
Duration of hospitalization (days)	13 (11-17)	14 (10-22)	0.553[c]
Day of 1st dose of TCZ administration	2 (2-3)	2 (2-3)	0.964[c]
day 1.	4 (4.7%)	2 (5.9%)	-
day 2.	50 (58.1%)	19 (55.9%)	-
day 3.	15 (17.4%)	6 (17.7%)	-
day 4.	8 (9.3%)	3 (8.8%)	-
after day 4.	9 (10.5%)	4 (11.7%)	-
Time from symptoms onset to TCZ (days)	12 (9-13)	10 (9-14)	0.337[c]
Time from dyspnoea onset to TCZ (days)	3 (2-6)	4,5 (3-7)	0.086[c]
Symptoms on admission			
cough	64 (74.4%)	23 (67.6%)	0.454[a]

subfebrile (37.5-38.3°C)	13 (15.1%)	2 (5.9%)	0.140[d]
fever (38.3-39.3°C)	65 (75.6%)	23 (67.7%)	0.376[a]
hyperpyrrexia (>39.3°C)	6 (7.0%)	2 (5.9%)	0.594[d]
dyspnoea at rest	47 (54.7%)	22 (64.7%)	0.315[a]
exertional dyspnoea only	19 (22.1%)	6 (17.7%)	0.589[a]
hypoxemia without dyspnoea	20 (23.3%)	6 (17.7%)	0.501[a]
malaise	44 (51.2%)	12 (35.3%)	0.116[a]
chest pain	7 (8.1%)	5 (14.7%)	0.224[d]
hemoptysis	2 (2.3%)	1 (2.9%)	0.636[d]
abdominal pain	4 (4.7%)	1 (2.9%)	0.562[d]
nausea	23 (26.7%)	7 (20.6%)	0.483[a]
vomiting	4 (4.7%)	2 (5.9%)	0.547[d]
diarrhoea	9 (10.5%)	4 (11.7%)	0.532[d]
headache	14 (16.3%)	5 (14.7%)	0.831[a]
myalgia	21 (24.4%)	9 (26.5%)	0.815[a]
arthralgia	5 (5.8%)	2 (5.9%)	0.640[d]
pharyngitis	2 (2.23%)	2 (5.9%)	0.318[d]
catarrh	14 (16.3%)	6 (17.7%)	0.856[a]
dysgeusia	16 (18.6%)	6 (17.7%)	0.902[a]
ageusia	9 (10.5%)	4 (11.8%)	0.532[d]
hypo- and anosmia	11 (12.8%)	2 (5.9%)	0.226[d]
Mental status (ACDU)			
A (Alert)	85 (98.8%)	32 (94.1%)	0.193[b]
C (Confused)	1 (1.2%)	2 (5.9%)	
Lung parenchyma involvement in CT (%)*	50 (35-60)	70 (60-85)	<0.001[c]
COVID-RRS*	5.0 (4.0-6.0)	7.3 (5.6-8.5)	<0.001[c]
Baseline PaO_2/FiO_2*	203 (136-277)	106 (80-177)	<0.001[c]
SpO_2/FiO_2 on TCZ initiation*	179 (120-239)	111 (102-152)	<0.001[c]
WHO clinical progression scale on TCZ initiation*			
5	75 (87.2%)	23 (67.6%)	0.013[a]
6	11 (12.7%)	11 (32.4%)	
Concomitant treatment:			
intravenous dexamethasone	86 (100%)	34 (100%)	-
remdesivir	71 (82.6%)	27 (79.4%)	0.688[a]
convalescent plasma	61 (70.9%)	24 (70.5%)	0.970[a]
antibiotics	25 (29.1%)	14 (41.2%)	0.212[a]
LMWH	84 (97.7%)	32 (94.1%)	0.318[d]
prophylaxic dose	38 (44.2%)	15 (44.1%)	0.994[a]
moderate dose	42 (48.8%)	16 (47.1%)	0.860[a]
therapeutic dose	4 (4.7%)	1 (2.9%)	0.562[d]
NOACs	4 (4.7%)	1 (2.9%)	0.562[d]

ASA	3 (3.5%)	3 (8.8%)	0.438[d]
$P_2Y_{12}i$	1 (1.2%)	-	0.717[d]
ACEI	24 (27.9%)	10 (29.4%)	0.869[a]
ARB	13 (15.1%)	7 (20.6%)	0.469[a]
CCB	20 (23.3%)	13 (38.2%)	0.097[a]
β-blockers	19 (22.1%)	12 (35.3%)	0.136[a]
diuretics	18 (20.9%)	10 (29.4%)	0.322[a]
flozins	3 (3.5%)	2 (5.9%)	0.438[d]
insulin	6 (7.0%)	3 (8.8%)	0.495[d]
GLP-1 agonists	2 (2.3%)	1 (2.9%)	0.636[d]
metformin	16 (18.6%)	7 (20.6%)	0.804[a]
DPP-4 inhibitors	1 (1.2%)	-	0.534[d]
sulfonylureas	5 (5.8%)	3 (8.8%)	0.406[d]
statins	28 (32.6%)	13 (38.2%)	0.555[a]
IS treatment	4 (4.7%)	1 (2.9%)	0.562[d]
DMARDs treatment	5 (5.8%)	2 (5.9%)	0.641[d]

*p<0,05 – [a]χ^2 test, [b]t-Student test, [c]Mann-Whitney U test or [d]Fisher's exact test, as appropriate COPD – chronic obstructive pulmonary disease, TCZ – tocilizumab, ACDU – mental status score (Alert, Confused, Drowsy, Unresponsive), CT – computed tomography, PaO_2 – partial pressure of arterial oxygen, FiO_2 – fraction of inspired oxygen, SpO_2 – peripheral oxygen saturation measured with pulse oximeter, WHO – World Health Organization, LMWH – low molecular weight heparin, NOACs – novel oral anticoagulants, ASA – acetylsalicylic acid, $P_2Y_{12}i$ – P_2Y_{12} inhibitors, ACEI – angiotensin converting enzyme inhibitors, ARB – angiotensin II receptor blockers, CCB – calcium channel blockers, GLP-1 – glucagon-like peptide 1, DPP-4 – dipeptidyl peptidase-4, IS – immunosuppressive, DMARDs – disease-modifying antirheumatic drugs
