# Supplementary material for: Identification of Clinical Response Predictors of Tocilizumab Treatment in Patients with Severe COVID-19 Based on Single-Center Experience
Source: J Clin Med. 2023 Mar 22;12(6):2429. doi: 10.3390/jcm12062429 (PMC10051490; doi:10.3390/jcm12062429)
Supplement: Supplementary file 1 [file jcm-12-02429-s001.zip › Table S3.pdf]

Journal of
Clinical Medicine

Supplementary Table S3. Extended clinical characteristics of study groups, presented as median (Q1-Q3). *P* values were derived with Mann-Whitney U test.

Parameter /time	Group	temperature (°C)	*P* value	SBP (mmHg)	value	DBP (mmHg)	P value
Baseline	CR	37.2 (36.7-38.3)	0.225	127 (120-146)	0.366	80 (70-85)	0.003
	NR	36.9 (36.6-37.8)		125 (118-138)		70 (63-80)	
On TCZ administration	CR	36.8 (36.6-37.1)	0.610	124 (118-138)	0.879	77 (70-82)	0.956
	NR	36.8 (36.6-37.1)		126 (119-137)		74 (70-86)	
On 2nd day after TCZ	CR	36.6 (36.5-36.7)	0.051	126 (115-140)	0.342	77 (70-83)	0.858
	NR	36.7 (36.6-37.1)		131 (119-140)		77 (70-85)	
On 5th day after TZ	CR	36.6 (36.5-36.8)	0.088	120 (114-133)	0.970	77 (70-84)	0.015
	NR	36.8 (36.6-37.1)		121 (11-137)		72 (66-77)	
Last recorded	CR	36.6 (36.6-36.8)	<0.001	125 (115-134)	0.497	77 (70-85)	0.409
	NR	36.8 (36.6-37.1)		120 (115-134)		76 (70-83)	

Parameter /time	Group	HR (/min)	*P* value	RR (/min)	*P* value	QO_2 (L/min)	*P* value
Baseline	CR	88 (78-100)	0.035	18 (15-20)	0.040	6 (4-15)	<0.001
	NR	79 (74-90)		20 (16-25)		14 (6-15)	
On TCZ administration	CR	78 (71-90)	0.863	16 (14-20)	<0.001	7.5 (5-15)	<0.001
	NR	79 (69-86)		21 (18-26)		15 (10-50)	
On 2nd day after TCZ	CR	70 (64-80)	0.051	14 (12-16)	<0.001	5 (3-12)	<0.001
	NR	72 (65-84)		25 (16-27)		50 (15-60)	
On 5th day after TZ	CR	74 (64-84)	0.322	13 (12-14)	<0.001	2 (0-5)	<0.001
	NR	75 (67-89)		20 (17-22)		50(45-60)	
Last recorded	CR	80 (70-90)	0.364	12 (12-13)	<0.001	0 (0-0)	<0.001
	NR	82 (73-92)		20 (12-20)		50 (0-50)	

**Parameter /time**	**Group**	**MEWS**	***P* value**	**qSOFA**	**value**	**WHO OS**	***P* value**
Baseline	CR	1 (1-2)	0.875	0 (0-1)	0.006	5 (5-5)	0.106
	NR	1 (1-3)		1 (0-1)		5 (5-5)	
On TCZ administration	CR	1 (0-2)	0.008	0 (0-1)	<0.001	5 (5-5)	0.013
	NR	2 (1-2)		1 (0-1)		5 (5-6)	
On 2nd day after TCZ	CR	0 (0-1)	<0.001	0 (0-0)	<0.001	5 (5-5)	<0.001
	NR	2 (0-3)		1 (0-1)		6 (5-6)	
On 5th day after TZ	CR	0 (0-0)	<0.001	0 (0-0)	<0.001	5 (4-5)	<0.001
	NR	2 (1-3)		1 (0-2)		6 (6-8)	
Last recorded	CR	0 (0-0)	<0.001	0 (0-0)	<0.001	4 (4-4)	<0.001
	NR	3 (0-3)		2 (0-2)		8 (4-9)	

**Parameter /time**	**Group**	**ROX index**	***P* value**	**HR-ROX index**	***P* value**	**SpO_2/FiO_2**	**P value**
Baseline	CR	11.1 (6.1-17.2)	0.006	12.9 (7.2-19.5)	0.027	202 (121-251)	0.007
	NR	6.5 (4.6-11.3)		7.2 (5.5-11.7)		121 (111-208)	
On TCZ administration	CR	10.9 (6.6-16.1)	<0.001	14.4 (7.2-21.3)	<0.001	179 (120-239)	<0.001
	NR	5.3 (4.0-7.7)		7.0 (4.8-11.3)		111 (102-152)	
On 2nd day after TCZ	CR	15.2 (9.7-21.7)	<0.001	20.6 (12.9-32.2)	<0.001	227 (142-287)	<0.001
	NR	4.8 (3.6-7.9)		6.8 (4.6-12.1)		114 (99-122)	
On 5th day after TZ	CR	24.1 (15.3-37.2)	<0.001	34.7 (19.6-47.8)	<0.001	324 (228-451)	<0.001
	NR	6.1 (4.9-7.0)		7.7 (6.0-9.8)		115 (101-130)	
Last recorded	CR	37.9 (35.6-38.5)	<0.001	46.6 (39.8-54.1)	<0.001	457 (448-462)	<0.001
	NR	5.6 (4.8-36.6)		7.7 (5.3-42.1)		112 (103-448)	

CR – clinical responder, NR – non-responder, SBP – systolic blood pressure, DBP – diastolic blood pressure, HR - heart rate, RR – respiratory rate, QO2 – oxygen flow, MEWS – Modified Early Warning Score, qSOFA - quick Sepsis Related Organ Failure Assessment score, WHO OS – World Health Organization Ordinal Scale, CT – computed tomography, FiO_2 – fraction of inspired oxygen, SpO_2 – peripheral oxygen saturation measured with pulse oximeter
