# Supplementary material for: Identification of Clinical Response Predictors of Tocilizumab Treatment in Patients with Severe COVID-19 Based on Single-Center Experience
Source: J Clin Med. 2023 Mar 22;12(6):2429. doi: 10.3390/jcm12062429 (PMC10051490; doi:10.3390/jcm12062429)
Supplement: Supplementary file 1 [file jcm-12-02429-s001.zip › Table S4.pdf]

Journal of
*Clinical Medicine*

**Supplementary Table S4.** Extended laboratory characteristics of studied groups and outcomes, presented as median (Q1-Q3). *P* values were derived with Mann-Whitney U test.

| Parameter /time | Group | $PaO_2$ (mmHg) | *P* value | $PaO_2$ /$FiO_2$ | *P* value | Hgb (mmol/L) | *P* value |
|---|---|---|---|---|---|---|---|
| Baseline | CR | 87 (73-1310 | <0.001 | 203 (126-277) | <0.001 | 8.5 (7.8-9.0) | 0.213 |
| | NR | 64 (61-78) | | 106 (78-177) | | 8.0 (7.6-9.0) | |
| On TCZ administration | CR | - | - | - | - | 8.3 (7.8-8.9) | 0.546 |
| | NR | - | | - | | 8.2 (7.6-8.8) | |
| On 2nd day after TCZ | CR | - | - | - | - | 8.3 (7.6-8.9) | 0.813 |
| | NR | - | | - | | 8.4 (7.6-9.1) | |
| On 5th day after TCZ | CR | - | - | - | - | 8.8 (7.9-9.4) | 0.610 |
| | NR | - | | - | | 8.8 (7.9-9.6) | |
| Last recorded | CR | - | - | - | - | 8.8 (8.1-9.5) | 0.098 |
| | NR | - | | - | | 8.5 (7.4-9.2) | |

| Parameter /time | Group | RBC (G/L) | *P* value | Hct (%) | *P* value | WBC (G/L) | *P* value |
|---|---|---|---|---|---|---|---|
| Baseline | CR | 4.7 (4.3-5.1) | 0.347 | 39.9 (36.7-42.4) | 0.142 | 6.6 (5.2-8.3) | 0.720 |
| | NR | 4.5 (4.1-4.9) | | 37.8 (36.1-41.5) | | 6.3 (4.7-9.5) | |
| On TCZ administration | CR | 4.5 (4.2-5.0) | 0.720 | 39.3 (36.8-42.6) | 0.566 | 5.5 (5.0-7.6) | 0.006 |
| | NR | 4.4 (4.1-4.9) | | 38.7 (35.5-42.0) | | 7.4 (5.5-9.8) | |
| On 2nd day after TCZ | CR | 4.5 (4.2-4.9) | 0.416 | 39.2 (36.0-42.4) | 0.495 | 6.0 (4.2-7.9) | <0.001 |
| | NR | 4.6 (4.1-5.0) | | 40.1 (36.0-42.9) | | 7.8 (7.0-11.2) | |
| On 5th day after TCZ | CR | 4.8 (4.3-5.1) | 0.275 | 41.3 (37.0-44.0) | 0.643 | 7.1 (5.4-8.4) | <0.001 |
| | NR | 4.9 (4.4-5.3) | | 41.3 (37.4-45.1) | | 12.2 (9.4-15.8) | |
| Last recorded | CR | 4.8 (4.3-5.3) | 0.322 | 41.7 (37.8-45.7) | 0.322 | 6.6 (4.9-9.7) | <0.001 |
| | NR | 4.7 (4.1-5.2) | | 40.2 (36.7-44.3) | | 11.8 (6.5-30.9) | |

| Parameter /time | Group | Neutrocytes (G/L) | *P* value | Monocytes (G/L) | *P* value | Lymphocytes (G/L) | *P* value |
|---|---|---|---|---|---|---|---|
| Baseline | CR | 5.1 (3.8-6.3) | 0.426 | 0.4 (0.2-0.5) | 0.350 | 0.7 (0.5-0.9) | 0.517 |
| | NR | 5.3 (3.4-8.7) | | 0.3 (0.2-0.5) | | 0.8 (0.5-0.9) | |
| On TCZ administration | CR | 4.2 (3.0-5.8) | 0.003 | 0.3 (0.2-0.4) | 0.388 | 0.8 (0.6-1.1) | 0.951 |
| | NR | 5.9 (4.4-8.0) | | 0.4 (0.2-0.6) | | 0.8 (0.6-1.0) | |
| On 2nd day after TCZ | CR | 4.2 (2.8-5.7) | <0.001 | 0.4 (0.3-0.7) | 0.286 | 1.1 (0.8-1.4) | 0.023 |
| | NR | 6.3 (5.4-8.8) | | 0.4 (0.2-0.6) | | 0.9 (0.6-1.1) | |
| On 5th day after TCZ | CR | 4.6 (3.4-6.5) | <0.001 | 0.6 (0.5-0.8) | 0.053 | 1.5 (1.1-2.2) | <0.001 |
| | NR | 9.5 (6.4-13.0) | | 0.5 (0.4-0.7) | | 0.9 (0/6-1.3) | |
| Last recorded | CR | 4.1 (2.6-6.5) | <0.001 | 0.7 (0.5-0.9) | 0.018 | 1.7 (1.2-2.5) | <0.001 |
| | NR | 9.3 (4.6-17.3) | | 0.6 (0.4-0.8) | | 1.2 (0.6-1.7) | |

| Parameter /time | Group | NLR | *P* value | NMR | *P* value | PLT (G/L) | P value |
|---|---|---|---|---|---|---|---|
| Baseline | CR | 6.0 (5.0-10.3) | 0.764 | 14.2 (9.5-21.2) | 0.235 | 204 (150-274) | 0.939 |
| | NR | 7.4 (4.7-10.8) | | 16.9 (10.9-31.2) | | 201 (166-254) | |
| On TCZ administration | CR | 5.0 (3.2-9.4) | 0.069 | 14.0 (7.9-21.9) | 0.287 | 237 (177-300) | 0.791 |
| | NR | 7.0 93.8-11.9) | | 15.0 (11.1-20.4) | | 224 (198-293) | |
| On 2nd day after TCZ | CR | 3.7 (2.3-5.5) | <0.001 | 8.9 (6.3-12.9) | <0.001 | 305 (221-368) | 0.278 |
| | NR | 7.6 (4.2-13.5) | | 16.3 (10.6-24.2) | | 275 (211-317) | |
| On 5th day after TCZ | CR | 3.1 (1.9-5.4) | <0.001 | 7.3 (5.4-10.3) | <0.001 | 343 (256-394) | 0.007 |
| | NR | 9.8 (6.2-19.7) | | 17.6 (11.5-32.1) | | 269 (184-350) | |
| Last recorded | CR | 2.3 (1.3-3.9) | <0.001 | 5.5 (3.2-8.0) | <0.001 | 312 (252-381) | <0.001 |
| | NR | 9.9 (3-24.1) | | 14.9 (6.9-32.0) | | 209 (151-297) | |

| Parameter /time | Group | INC (G/L) | *P* value | CRP (mg/L) | *P* value | Natremia (mmol/L) | *P* value |
|---|---|---|---|---|---|---|---|
| Baseline | CR | 0.04 (0.02-0.06) | 0.426 | 118.5 (66.7-165.7) | 0.809 | 138 (135-139) | 0.107 |
| | NR | 0.04 (0.02-0.06) | | 129.8 (62.4-189.5) | | 136 (132-139) | |
| On TCZ administration | CR | 0.04 (0.02-0.07) | 0.051 | 111.9 (66.1-185.6) | 0.643 | 139 (137-141) | 0.465 |
| | NR | 0.06 (0.04-0.10) | | 101.2 (64.3-166) | | 139 (137-141) | |
| On 2nd day after TCZ | CR | 0.05 (0.03-0.12) | 0.002 | 46.0 (21.7-83.9) | 0.428 | 140 (139-142) | 0.771 |
| | NR | 0.12 (0.05-0.33) | | 41.7 (20.0-59.6) | | 141 (138-143) | |
| On 5th day after TCZ | CR | 0.06 (0.03-0.14) | 0.007 | 9.6 (4.6-19.8) | 0.775 | 139 (137-142) | 0.853 |
| | NR | 0.14 (0.05-0.29) | | 8.7 (4.5-23.7) | | 139 (139-141) | |
| Last recorded | CR | 0.07 (0.02-0.17) | <0.001 | 2.9 (1.1-5.5) | 0.004 | 139 (137-141) | <0.001 |
| | NR | 0.28 (0.06-0.86) | | 8.1 (1.0-71.9) | | 142 (139-146) | |

| Parameter /time | Group | Kaliemia (mmol/L) | *P* value | SCr (μmol/L) | *P* value | BUN (mg/dL) | P value |
|---|---|---|---|---|---|---|---|
| Baseline | CR | 4.1 (3.8-4.4) | 0.308 | 74 (65-89) | 0.030 | 15.4 (12.3-21.5) | 0.018 |
| | NR | 4.2 (3.9-4.5) | | 85 (73-109) | | 20.5 (15.7-22.9) | |
| On TCZ administration | CR | 4.3 (4.0-4.7) | 0.757 | 69 (61-81) | 0.068 | 16.3 (13.2-21.8) | 0.003 |
| | NR | 4.4 (4.0-4.7) | | 76 (68-91) | | 22.7 (15.3-25.2) | |
| On 2nd day after TCZ | CR | 4.5 (4.2-4..8) | 0.508 | 67 (61-76) | 0.056 | 17.0 (13.8-21.8) | 0.005 |
| | NR | 4.4 (4.2-4.7) | | 72 (64-85) | | 23.8 (15.1-31.8) | |
| On 5th day after TCZ | CR | 4.6 (4.2-4.8) | 0.443 | 68 (62-79) | 0.005 | 16.0 (13.7-20.8) | <0.001 |
| | NR | 4.5 (4.0-4.9) | | 80 (64-108) | | 25.5 (18.7-34.6) | |
| Last recorded | CR | 4.5 (4.2-4.8) | 0.267 | 70 (62-83) | <0.001 | 15.7 (12.3-19.5) | <0.001 |
| | NR | 4.6 (4.2-5.1) | | 99 (72-160) | | 35.1 (18-61.3) | |

| Parameter /time | Group | Glucose (mmol/L) | *P* value | hs-TnI (ng/L) | *P* value | Total bilirubin ( µmol/L) | *P* value |
|---|---|---|---|---|---|---|---|
| Baseline | CR | 5.9 (5.2-7.3) | 0.052 | 4 (4-8) | <0.001 | 8.4 (6.7-11.4) | 0.641 |
| | NR | 6.7 (5.9-8.1) | | 11 (5-24) | | 7.8 (6.5-11.2) | |
| On TCZ administration | CR | - | - | 3 (3-6) | <0.001 | 8.9 (6.0-11.0) | 0.433 |
| | NR | - | | 11 (4-31) | | 8.0 (6.4-9.6) | |
| On 2nd day after TCZ | CR | - | - | - | - | 8.9 (6.1-11.5) | 0.435 |
| | NR | - | | - | | 8.3 (7.1-9.3) | |
| On 5th day after TCZ | CR | - | - | 3 (3-4) | ,<0.001 | 10.9 (8.3-13.0) | 0.789 |
| | NR | - | | 18 (4-101) | | 11.2 (8.0-13.1) | |
| Last recorded | CR | - | - | 3 (3-4) | ,<0.001 | 10.4 (8.8-13.7) | 0.508 |
| | NR | - | | 15 (4-201) | | 11.6 (9.3-14.5) | |

| Parameter /time | Group | AST (IU/L) | *P* value | ALT (IU/L) | *P* value | ALP (IU/L) | *P* value |
|---|---|---|---|---|---|---|---|
| Baseline | CR | 42 (31-59) | 0.038 | 38 (27-54) | 0.171 | - | - |
| | NR | 52 (35-76) | | 32 (19-50) | | - | |
| On TCZ administration | CR | 42 (32-59) | 0.085 | 43 (29-67) | 0.243 | 61 (48-72) | 0.071 |
| | NR | 47 (38-78) | | 35 (24-62) | | 71 (50-81) | |
| On 2nd day after TCZ | CR | 41 (31-57) | 0.166 | 58 (37-85) | 0.039 | - | - |
| | NR | 46 (36-70) | | 44 (26-65) | | - | |
| On 5th day after TCZ | CR | 41 (27-66) | 0.359 | 73 (50-125) | 0.039 | 61 (47-68) | <0.001 |
| | NR | 47 (28-100) | | 52 (35-98) | | 86 (64-93) | |
| Last recorded | CR | 35 (26-52) | 0.051 | 89 (48-128) | 0.139 | 60 (54-65) | <0.001 |
| | NR | 44 (27-126) | | 63 (38-106) | | 88 (59-108) | |

| Parameter /time | Group | GGT (IU/L) | *P* value | INR | *P* value | aPTT (s) | *P* value |
|---|---|---|---|---|---|---|---|
| Baseline | CR | - | - | 1.16 (1.09-1.24) | 0.981 | 32.8 (29.3-37.1) | 0.525 |
| | NR | - | | 1.16 (1.10-1.22) | | 33.4 (30.3-36.4) | |
| On TCZ administration | CR | 76 (36-138) | 0.596 | 1.15 (1.08-1.30) | 0.519 | 32.6 (28.8-36.2_ | 0.017 |
| | NR | 58 (36-113) | | 1.15 (1.08-1.25) | | 28.1 (25.5-30.8) | |
| On 2nd day after TCZ | CR | - | - | - | - | - | - |
| | NR | - | | - | | - | |
| On 5th day after TCZ | CR | 81 (38-162) | 0.999 | 1.10 (1.04-1.19) | ,0.058 | 28.1 (25.5-30.8) | 0.921 |
| | NR | 90 (40-148) | | 1.13 (1.09-1.24) | | 28.4 (24.7-31.1) | |
| Last recorded | CR | 90 (53-131) | 0.896 | 1.11 (1.04-1.48) | ,0.672 | 27.5 (24.8-30.5) | 0.208 |
| | NR | 85 (44-137) | | 1.17 (1.08-1.31) | | 28.6 (24.8-33.7) | |

| Parameter /time | Group | D-Dimer (μg/mL) | *P* value | TSH (μIU/L) | *P* value | 25(OH)$D_3$ (ng/mL) | *P* value |
|---|---|---|---|---|---|---|---|
| Baseline | CR | 0.8 (0.6-1.4) | 0.552 | 0.91 (0.60-1.20) | 0.104 | 27 (17-34) | 0.013 |
| | NR | 0.9 (0.6-1.3) | | 0.62 (0.32-1.11) | | 24 (13-28) | |
| On TCZ administration | CR | 0.9 (0.6-1.5) | 0.005 | - | - | - | - |
| | NR | 1.7 (0.7-3.7) | | - | | - | |
| On 2nd day after TCZ | CR | 1.1 (0.7-1.8) | <0.001 | - | - | - | - |
| | NR | 6.1 (1.4-27.4) | | - | | - | |
| On 5th day after TCZ | CR | 1.2 (0.6-1.7) | <0.001 | - | - | - | - |
| | NR | 6.5 (1.7-20.1) | | - | | - | |
| Last recorded | CR | 0.7 (0.4-1.4) | <0.001 | - | - | - | - |
| | NR | 2.2 (1.5-4.8) | | - | | 8- | |

| Parameter /time | Group | TP (g/L) | *P* value | albumin (g/L) | *P* value | ferritin (ng/mL) | *P* value |
|---|---|---|---|---|---|---|---|
| Baseline | CR | - | - | - | - | - | - |
| | NR | - | | - | | - | |
| On TCZ administration | CR | 63 (60-66) | 0.518 | 36 (34-38) | 0.335 | 1144 (697-1527) | 0.849 |
| | NR | 62 (59-66) | | 36 (33-37) | | 1075(646-1737) | |
| On 2nd day after TCZ | CR | - | - | - | - | - | - |
| | NR | - | | - | | - | |
| On 5th day after TCZ | CR | 63 (59-66) | 0.029 | 37 (34-40) | ,<0.001 | 1156 (633-1789) | 0.012 |
| | NR | 59 (56-63) | | 33 (30-37) | | 800 (508-1106) | |
| Last recorded | CR | - | - | - | ,- | 590 (439-706) | <0.001 |
| | NR | - | | - | | 1291 (784-1719) | |

| Parameter /time | Group | LDH (IU/L) | *P* value | Chloride (mmol/L) | *P* value | CK (IU/L) | *P* value |
|---|---|---|---|---|---|---|---|
| Baseline | CR | 405 (326-485) | <0.001 | - | - | - | - |
| | NR | 510 (417-695) | | - | | - | |
| On TCZ administration | CR | 408 (355-478) | <0.001 | 102 (100-104) | 0.175 | 104 (56-188) | 0.031 |
| | NR | 646 (463-802) | | 104 (100-105) | | 167 (79-371) | |
| On 2nd day after TCZ | CR | 374 (312-444) | <0.001 | - | - | - | - |
| | NR | 610 (486-871) | | - | | - | |
| On 5th day after TCZ | CR | 332 (272-408) | <0.001 | 103 (102-106) | 0.858 | 41.5 (27-61) | <0.001 |
| | NR | 743 (477-961) | | 104 (102-105) | | 100 (47-194) | |
| Last recorded | CR | 258 (229-333) | <0.001 | - | - | - | - |
| | NR | 715 (323-977) | | - | | 8- | |

| **Parameter /time** | **Group** | **BNP (ng/L)** | ***P* value** | **PCT (ng/mL)** | ***P* value** |
|---|---|---|---|---|---|
| Baseline | CR | - | - | 0.08 (0.05-0.15) | 0.645 |
| | NR | - | | 0.08 (0.05-0.18) | |
| On TCZ administration | CR | 32 (16-55) | 0.003 | 0.07 (0.05-0.14) | 0.464 |
| | NR | 61 (38-103) | | 0.09 (0.04-0.24) | |
| On 2nd day after TCZ | CR | - | - | 0.04 (0.03-0.08) | 0.418 |
| | NR | - | | 0.05 (0.03-0.10) | |
| On 5th day after TCZ | CR | 19 (8-40) | 0<0.001 | 0.03 (0.02-0.04) | ,0.015 |
| | NR | 86 (36-134) | | 0.04 (0.02-0.14) | |
| Last recorded | CR | - | - | 0.02 (0.02.-0.02) | <0.001,- |
| | NR | - | | 0.30 (0.02-1.38) | |

$PaO_2$ – partial presssure of arterial oxygen, $FiO_2$ – fraction of inspired oxygen, Hgb – hemoglobin, RBC – red blood cell count, Hct – hematocrit, WBC – white blood cell count, NLR – neutrocyte-to-lymphocyte ratio, NMR – neutrocyte-to-monocyte ratio, PLT – platelet count, INC – immature neutrocyte count, CRP – C-reactive protein concentration, SCr – serum creatinine concentration, BUN – blood urea nitrogen, hs-TnI – high sensitive troponin I, AST – aspartate aminotransferase, ALT – alanine aminotransferase, ALP – alkaline phosphatase, GGT – gamma-glutamyl transferase, INR – international normalized ratio, aPTT - activated partial thromboplastin time, TSH – thyrotropin, 25(OH)$D_3$ - calcifediol, TP – total protein, LDH – lactate dehydrogenase, CK - creatine kinase , BNP – B-type natriuretic peptide, PCT - procalcitionin
