# Supplementary material for: Identification of Clinical Response Predictors of Tocilizumab Treatment in Patients with Severe COVID-19 Based on Single-Center Experience
Source: J Clin Med. 2023 Mar 22;12(6):2429. doi: 10.3390/jcm12062429 (PMC10051490; doi:10.3390/jcm12062429)
Supplement: Supplementary file 1 [file jcm-12-02429-s001.zip › Table S5.pdf]

Journal of
Clinical Medicine

MDPI

Supplementary Table S5. Outcomes and their prevalence upon administration and occurrence after treatment with tocilizumab in studied groups.

Outcome	Prevalence upon administration			Occurence after administration		
	Clinical responders	Non-responders	*P* value	Clinical responders	Non-responders	*P* value
death*	-	-	-	-	20 (58.8%)	<0.001[a]
ICU transfer due to ARDS progression*	-	-	-	-	20 (58.8%)	<0.001[a]
PE	2 (2.3%)	2 (5.9%)	0.317[b]	1 (1.2%)	3 (8.8%)	0.068[b]
DVT	-	-		2 (2.3%)	-	0.511*
CAC*	-	2 (5.9%)	0.078[b]	31 (36.1%)	22 (64.7%)	0.004[a]
DIC	-	-	-	-	1 (2.9%)	0.283[b]
severe lymphopenia	28 (32.6%)	13 (38.2%)	0.554[a]	21 (24.4%)	11 (32.4%)	0.376[a]
haemolytic anemia	-	-	-	1 (1.2%)	3 (8.8%)	0.068[b]
neutropenia	15 (17.4%)	3 (8.8%)	0.233[a]	13 (15.1%)	2 (5.9%)	0.140[b]
agranulocytosis	-	-	-	10 (11.6%)	2 (5.9%)	0.282[b]
thrombocytopenia	29 (33.7%)	9 (26.5%)	0.441[a]	10 (11.6%)	9 (26.5%)	0.045[a]
severe thrombocytopenia	-	-	-	6 (7.0%)	5 (14.7%)	0.165[b]
major bleeding	-	-	-	-	1 (2.9%)	0.283[b]
minor bleeding	-	-	-	3 (3.5%)	6 (17.7%)	0.015[b]
MACE	8 (9.3%)	11 (32.4%)	0.001[a]	3 (3.5%)	9 (26.5%)	<0.001[b]
AMI	7 (8.1%)	11 (32.4%)	<0.001[a]	2 (2.3%)	8 (23.5%)	<0.001[b]
ACS	-	-	-	-	1 (2.9%)	0.283[b]
myocarditis	1 (1.2%)	-	0.716[b]	-	-	-
new onset of AF	1 (1.2%)	-	0.716[b]	1 (1.2%)	-	0.716[b]
HF decompensation	1 (1.2%)	-	0.716[b]	1 (1.2%)	-	0.716[b]
liver injury	28 (32.6%)	9 (26.5%)	0.515[a]	18 (20.9%)	15 (44.1%)	0.010[a]
cholestatic	27 (31.4%)	7 (20.6%)	0.236[a]	8 (9.3%)	5 (14.7%)	0.289[b]
hepatocellular	1 (1.2%)	2 (5.9%)	0.193[b]	5 (5.8%)	5 (14.7%)	0.113[b]
mixed	5 (5.8%)	3 (8.8%)	0.406[b]	11 (12.8%)	5 (14.7%)	0.494[b]
DILI	-	-	-	1 (1.2%)	-	0.716[b]
SLD	-	-	-	-	6 (17.7%)	<0.001[b]
patients with secondary infections	2 (2.3%)	4 (11.8%)	0.033[a]	6 (7.0%)	10 (29.4%)	0.002[b]
UTI	1 (1.2%)	1 (2.9%)	0.488[b]	4 (4.7%)	6 (17.7%)	0.029[b]
VAP (*A. baumanii*)	-	-	-	-	6 (17.7%)	<0.001[b]

bacteriaemia	-	-	-	2 (2.3%)	8 (23.5%)	<0.001[b]
sepsis	-	-	-	-	8 (23.5%)	<0.001[b]
septic shock	-	-	-	-	5 (14.7%)	0.001[b]
sinusitis	-	2 (5.9%)	0.078[b]	-	-	-
otitis media	1 (1.2%)	1 (1.2%)	0.488[b]	-	-	-
candidiasis	-	-	-	-	2 (5.9%)	0.078[b]
S. pneumoniae pneumonia	-	-	-	5 (5.8%)	3 (8.8%)	0.406[b]
L. pneumonophila pneumonia	-	-	-	-	2 (5.9%)	0.078[b]
AKI	-	-	-	6 (7.0%)	17 (50%)	<0.001[a]
prerenal	-	-	-	5 (5.8%)	11 (32.4%)	<0.001[b]
renal	-	-	-	1 (1.2%)	6 (17.7%)	<0.001[b]
AKI demanding RRT	-	-	-	-	13 (38.2%)	<0.001[b]
acute tubulointerstitial nephritis	6 (7.0%)	-	0.182[b]	4 (4.7%)	2 (5.9%)	0.547[b]
proteinuria	40 (46.5%)	15 (44.1%)	0.812[a]	2 (2.3%)	9 (26.5%)	<0.001[b]
sterile pyuria	34 (39.5%)	20 (58.8%)	0.056[a]	5 (5.8%)	9 (26.5%)	0.003[b]
acute confusional state	1 (1.2%)	2 (5.9%)	0.193[b]	-	-	-
COVID-19 encephalopathy	-	-	-	-	2 (5.9%)	0.078[b]
hypotension	-	1 (2.9%)	0.283[b]	9 (10.5%)	2 (5.9%)	0.726[b]
haemodynamic instability	-	-	-	6 (7.0%)	3 (8.8%)	0.711[b]
respiratory deterioration	-	-	-	13 (15.1%)	26 (76.5%)	<0.001[a]
COV-HI	50 (58.1%)	23 (67.7%)	0.336[a]	8 (9.3%)	14 (41.2%)	<0.001[a]
cHIS	65 (75.6%)	30 (88.3%)	0.124[a]	4 (4.7%)	3 (8.8%)	0.313[b]
CCS	10 (11.6%)	13 (38,2%)	<0.001[a]	4 (4.7%)	3 (8.8%)	0.313[b]

*P<0.05 - [a] χ^2 test or [b] Fisher's exact test.

ICU – intensive care unit, PE – pulmonary embolism, DVT – deep vein thrombosis, CAC – COVID-19 associated coagulopathy, DIC – disseminated intravascular coagulation, MACE – major adverse cardiovascular event, AMI – acute myocardial injury, ACS – acute coronary syndrome, AF – atrial fibrillation, HF – heart failure, DILI – drug-induced liver injury, SLD – severe liver dysfunction, UTI – urinary tract infection, VAP – ventilator-associated pneumonia, AKI – acute kidney injury, RRT – renal replacement therapy, COV-HI – COVID-19 hyperinflammation syndrome, cHIS - COVID-19-associated Hyperinflammation Syndrome score, CCS - COVID-19 cytokine storm.
